# Global gene expression analysis in etiolated and de-etiolated seedlings in conifers

**DOI:** 10.1371/journal.pone.0219272

**Published:** 2019-07-05

**Authors:** Sonali Sachin Ranade, Nicolas Delhomme, M. Rosario García-Gil

**Affiliations:** 1 Department of Forest Genetics and Plant Physiology, Umeå Plant Science Centre, Swedish University of Agricultural Sciences, SE, Umeå, Sweden; 2 Department of Plant Physiology, Umeå Plant Science Centre, Umeå University, SE, Umeå, Sweden; Institute of Genetics and Developmental Biology Chinese Academy of Sciences, CHINA

## Abstract

Plant life cycle begins with germination of seed below the ground. This is followed by seedling’s development in the dark: skotomorphogenesis; and then a light-mediated growth: photomorphogenesis. After germination, hypocotyl grows rapidly to reach the sun, which involves elongation of shoot at the expense of root and cotyledons. Upon reaching ground level, seedling gets exposed to sunlight following a switch from the etiolated (skotomorphogenesis) to the de-etiolated (photomorphogenesis) stage, involving a series of molecular and physiological changes. Gymnosperms have evolved very differently and adopted diverse strategies as compared to angiosperms; with regards to response to light quality, conifers display a very mild high-irradiance response as compared to angiosperms. Absence of apical hook and synthesis of chlorophyll during skotomorphogenesis are two typical features in gymnosperms which differentiate them from angiosperms (dicots). Information regarding etiolation and de-etiolation processes are well understood in angiosperms, but these mechanisms are less explored in conifer species. It is, therefore, interesting to know how similar these processes are in conifers as compared to angiosperms. We performed a global expression analysis (RNA sequencing) on etiolated and de-etiolated seedlings of two economically important conifer species in Sweden to review the differentially expressed genes associated with the two processes. Based on the results, we propose that high levels of *HY5* in conifers under DARK condition coupled with expression of few other genes associated with de-etiolation in angiosperms e.g. *SPA*, *DET1* (lower expression under DARK) and *CRY1* (higher expression under DARK), leads to partial expression of photomorphogenic genes in the DARK phenotype in conifers as displayed by absence of apical hook, opening of cotyledons and synthesis of chlorophyll.

## Introduction

Plants undergo different developmental mechanisms under dark and light conditions; skotomorphogenesis is the development under dark and photomorphogenesis is the light-mediated growth, and the switch from skotomorphogenic to photomorphogenic stage involves series of molecular and physiological changes. Skotomorphogenesis involves allocation of the resources towards hypocotyl elongation to reach the sunlight, at the expense of cotyledon and root development.

Phytochrome interacting factors (PIFs), encoded by a subset of the basic helix-loop-helix (bHLH) transcription factor superfamily, are the key modulators in maintaining the skotomorphogenic state in the dark by repressing the genes responsible for de-etiolation and also regulate the switch to photomorphogenesis [1, 2]. *COP*, *DET* and *FUS* are members of a group of genes termed as *CONSTITUTIVE PHOTOMORPHOGENIC/DE-ETIOLATED/*FUSCA (*COP/DET/FUS*) and, they encode proteins (e.g. COP1, DET1-3, COP10 and COP9 Signalosome (CSN) subunits) that are essential for suppressing photomorphogenesis and maintaining the etiolated state of seedlings in darkness. *Arabidopsis thaliana* (*A*. *thaliana*) *cop/det* mutants display light-grown phenotypes in darkness and *fus* mutants accumulate high levels of anthocyanin [3]. COP/DET/FUS proteins are constituents of three protein complexes: COP1/SUPPRESSOR OF PHYA 105 1 (SPA1) complex, the CSN and the CDD complex; all complexes are involved in the ubiquitin-mediated proteolytic degradation of photomorphogenesis-promoting factors [4–6]. The COP1/SPA1 E3 ubiquitin ligase is a repressor of photomorphogenesis that targets photomorphogenesis-promoting factors for proteolysis. The CSN is a conserved multi-protein complex (protease) that functions in the ubiquitin–proteasome pathway, which consists of six of the COP/DET/FUS proteins, with eight distinct subunits that regulates all CULLIN-RING E3 ligases (CRL) [3]. DET1 forms the CDD complex by interacting with DAMAGED DNA BINDING PROTEIN 1 (DDB1) and COP10; CDD complex physically associates with CULLLIN4 (CUL4) to form an E3 ligase that degrades light signal-promoting transcription factors to repress photomorphogenesis in darkness. COP1 mediates the degradation of several photomorphogenesis-promoting transcription factors (e.g. ELONGATED HYPOCOTYL 5—HY5) in dark, thus acts as a light-inactivable repressor of photomorphogenic development. HY5 is a bZIP transcription factor, which not only positively regulates photomorphogenic development but also regulates transcription of a large number of genes and plays a key role in the seedling development including fundamental developmental processes such as cell elongation and proliferation, chloroplast development and pigment accumulation, and also other signaling cascades such as hormonal and nutritional pathway [7]. COP1 negatively regulates *HY5* by directly interacting with HY5 [8]. Light activates the photoreceptors that inhibit the COP1/SPA1 complex [9], trigger the nuclear exclusion of COP1 and target PIFs leading to depletion of PIF levels. Exclusion of COP1 from nucleus along with the PIF degradation signal suppresses the skotomorphogenic genes and promotes activation of genes responsible for de-etiolation (photomorphogenesis).

Gymnosperms have adopted strategies which are different from angiosperms with reference to growth, development and maintenance. Dark germinated Scots pine seedlings lack the formation of apical hook which is a typical characteristic in most dicots germinated in dark or under soil [10, 11]. Furthermore, among the gymnosperms, conifers possess the ability to synthesize chlorophyll in the dark due to the presence of three genes (light-independent protochlorophyllide oxidoreductase subunits L, N and B—*chlL*, *chlN*, and *chlB*) in the chloroplast genome coding for subunits of the dark-operative, light-independent protochlorophyllide oxidoreductase (DPOR) involved in reduction of protochlorophyllide to chlorophyllide [12–15]. Etiolation and de-etiolation processes are well understood in angiosperms to a vast extend, but the information regarding genes involved in these processes with reference to conifer species remains sparse. In the current work, we performed a global expression analysis of the etiolated and de-etiolated seedlings of two economically important conifer species in Sweden to review the differentially expressed genes associated with the etiolation and de-etiolation process. The role of red (R) and far-red (FR) light in the phytochrome-mediated de-etiolation has been well characterized in *A*. *thaliana* [16, 17]. Furthermore, from our previous work [18, 19] and also the experiments performed by Clapham et al. [20, 21], it is now well established that pine and spruce respond to R and FR light wavelengths in a different way than angiosperms. Therefore, in our current work we have focused on the effect of R and FR light wavelengths on the process of de-etiolation and have considered a R to FR ratio which is a sun-like condition (R:FR 1.2). In this study we report the differential expression between the etiolated and de-etiolated seedlings in pine and spruce, grown under continuous dark and sun-like conditions, which were harvested at the same developmental stage (fully developed hypocotyl with opening of cotyledons as described by Ranade & García-Gil [18]) and used for gene expression analyses.

## Material and methods

### Seed material

Scots pine (*Pinus sylvestris* L.) and Norway spruce (*Picea abies* (L.) Karst.) seeds were collected from natural populations in Sweden (Scots pine: Kaunisvaara 67^o^5´N, 22^o^3´E; Norway spruce: Pellonhuhta 67^o^2´N, 22^o^1´E). Seeds were provided by Skogforsk, Forestry Research Institute of Sweden, which granted the authorization for using the seeds for the current study; no other permission was required for the study. This study does not involve protected or endangered species. A minimum distance of 50 meters was maintained between the selected trees to ensure low consanguinity. Cones were dried with warm air to force release the seeds. Sound seeds were separated from the empty seeds by floatation. The percentage of germination was obtained by sowing soaked seeds on paper discs on a warm bench with controlled humidity and temperature. The percentage of germination was 98% from a batch of 200 seeds (5 seeds per tree).

### Seed germination and light treatment

Stratified seeds (soaked in water at 4°C overnight) were sown on water-saturated sterile vermiculite in growth boxes (Saveen Werner) and maintained at a constant temperature of 22°C in Percival (LED-30 Elite series) growth cabinets. 70 sound seeds per treatment and per species (one seed per sampled tree) were germinated under continuous SUN (sun-like condition: R:FR 1.2; PAR 65 μmol m^-2^s^-1^; R = 35 μmol m^-2^s^-1^; FR = 30 μmol m^-2^s^-1^) and continuous DARK conditions. Seedlings were harvested at same developmental stage when the hypocotyl was fully developed [18].

### Transmission Electron Microscopy (TEM)

Cotyledons from three DARK grown Scots pine seedlings at the developmental stage with fully opened cotyledons were fixed with 2.5% glutaraldehyde in 0.1 M sodium cacodylate buffer. Samples were washed with buffer and post fixed in 1% osmium tetroxide, dehydrated with ethanol and prolpylene oxide and finally embedded in Spurr resin according to standard procedures [22]. Sections were contrasted with uranyl acetate and lead citrate, and examined with a JEM1230 transmission electron microscope (JEOL, Sollentuna, Sweden) operating at 80kV. Micrographs were acquired with a Gatan Orius 830 2kx2k CCD camera (Gatan, Abingdon, Great Britain) using Digital micrograph software.

### RNA sequencing (RNA-Seq)

Three experimental replicates were prepared for each of the light treatment (SUN and DARK) for RNA extraction for Scots pine and Norway spruce. Whole seedlings were used for RNA-Seq. The experimental replicates were prepared by pooling three seedlings per sample to reduce variation between replicates and to increase the statistical power of the analysis.

Total RNA was isolated using Spectrum Plant Total RNA Kit (Sigma) following the manufacturer’s instructions. The mRNA concentration (A260/280 ~ 2.0) and quality (RIN > 7) was determined using NanoDrop-2000 spectrophotometer (Thermo Fisher Scientific Inc.) and Bioanalyzer 2100 (Agilent Technologies Inc.), respectively. RNA library preparation and subsequent sequencing were performed at SciLifeLab (Stockholm, Sweden). Strand-specific RNA libraries for sequencing were prepared with TruSeq Stranded mRNA Sample prep kit of 96 dual indexes (Illumina) according to the manufacturer’s instructions except for the following changes: the protocols were automated in Agilent NGS workstation (Agilent Technologies) using purification steps as described earlier [23, 24]. Clonal clusters were generated using cBot (Illumina) and sequenced on HiSeq2500 (Illumina) according to manufacturer's instructions. Bcl to Fastq conversion was performed with bcl2Fastq v1.8.3 from the CASAVA software suite. The quality scale was Sanger / phred33 / Illumina 1.9. An initial nine samples were sequenced for 126 cycles, yielding an average of 68M PE reads / sample. Three additional samples were subsequently sequenced (replicate 4 in Table E in S1 File) yielding an average of 22M PE reads / sample. The data obtained was deposited to the ENA and is accessible under the accession number RJEB19683.

### Pre-processing of RNA-Seq data and differential expression analyses

The data pre-processing was performed as described here: http://www.epigenesys.eu/en/protocols/bio-informatics/1283-guidelines-for-rna-seq-data-analysis. Briefly, the quality of the raw sequence data was assessed using FastQC (http://www.bioinformatics.babraham.ac.uk/projects/fastqc/). Residual ribosomal RNA (rRNA) contamination was assessed and filtered using SortMeRNA (v2.1 [25]; settings—log—paired_in—fastx—sam—num_alignments 1) using the rRNA sequences provided with SortMeRNA (rfam-5s-database-id98.fasta, rfam-5.8s-database-id98.fasta, silva-arc-16s-database-id95.fasta, silva-bac-16s-database-id85.fasta, silva-euk-18s-database-id95.fasta, silva-arc-23s-database-id98.fasta, silva-bac-23s-database-id98.fasta and silva-euk-28s-database-id98.fasta). Data were then filtered to remove adapters and trimmed for quality using Trimmomatic (v0.32 [26]; settings TruSeq3-PE-2.fa:2:30:10 SLIDINGWINDOW:5:20 MINLEN:50). After both filtering steps, FastQC was run again to ensure that no technical artefacts were introduced. Filtered reads were aligned to v1.0 of the *P*. *abies* genome (retrieved from the PopGenIE resource, [27]) using STAR (v2.4.0f1 [28]; non default settings:—outSAMstrandField intronMotif—readFilesCommand zcat—outSAMmapqUnique 254—quantMode TranscriptomeSAM—outFilterMultimapNmax 100—outReadsUnmapped Fastx—chimSegmentMin 1—outSAMtype BAM SortedByCoordinate—outWigType bedGraph—alignIntronMax 11000). The annotations obtained from the *P*. *abies* v1.0 GFF file contain only one transcript per gene-model, and as such, did not need to be modified to generate ‘synthetic’ gene models. This GFF file and the STAR read alignments were used as input to the HTSeq [29] htseq-count python utility to calculate exon-based read count values. The htseq-count utility takes only uniquely mapping reads into account. A total of 46,916 genes were expressed in any of the condition (66.32% of all genes). The Scots pine samples were processed similarly, but aligned to the v1.01 of the *P*. *taeda* genome [30] and its annotation retrieved from http://pinegenome.org/pinerefseq/. A total of 37.6% genes were expressed in any of the condition (31, 764 of all genes). The biological relevance of the data—e.g. biological replicates similarity—was assessed by Principal Component Analysis (PCA) and other visualisations (e.g. heatmaps), using custom R scripts.

#### Differential expression analysis

Statistical analysis of single-gene differential expression was performed in R (v3.3.0 [31]) using the Bioconductor (v3.3 [32]) DESeq2 package (v1.12.0 [33]). FDR adjusted *P-*values were used to assess significance; a common threshold of 1% was used throughout and only genes with log_2_ fold change values -0.5> log_2_ fold change >0.5 were considered. For the data quality assessment (QA) and visualisation, the read counts were normalised using a variance stablishing transformation as implemented in DESeq2.

Our data analysis involves annotations retrieved from ConGenIE [34], which are based on the gene family analysis conducted in Norway spruce [35], where the gene family annotations were obtained using five angiosperms: *A*. *thaliana*, *Populus trichocarpa* Torr. & A. Gray ex Hook, *Vitis vinifera* L., *Oryza sativa* L. and *Zea mays* L., and two basal plants: *Selaginella moellendorffii* and *Physcomitrella patens* (Hedw.) Bruch & Schimp. Due to the limited manually curated annotation resources available for conifers, our results are interpreted based on the knowledge presented in the literature with reference to the metabolic pathways and mechanisms involved in etiolation/de-etiolation.

## Results

In this study, we have conducted an expression analysis associated with etiolation/de-etiolation in Scots pine and Norway spruce, and also compared the results to the well characterized angiosperm model system *A*. *thaliana*. Our work represents the first attempt to identify the genes involved in etiolation and de-etiolation processes in conifers.

### Morphological characteristics of the etiolated seedlings in conifers

**Fig 1:**
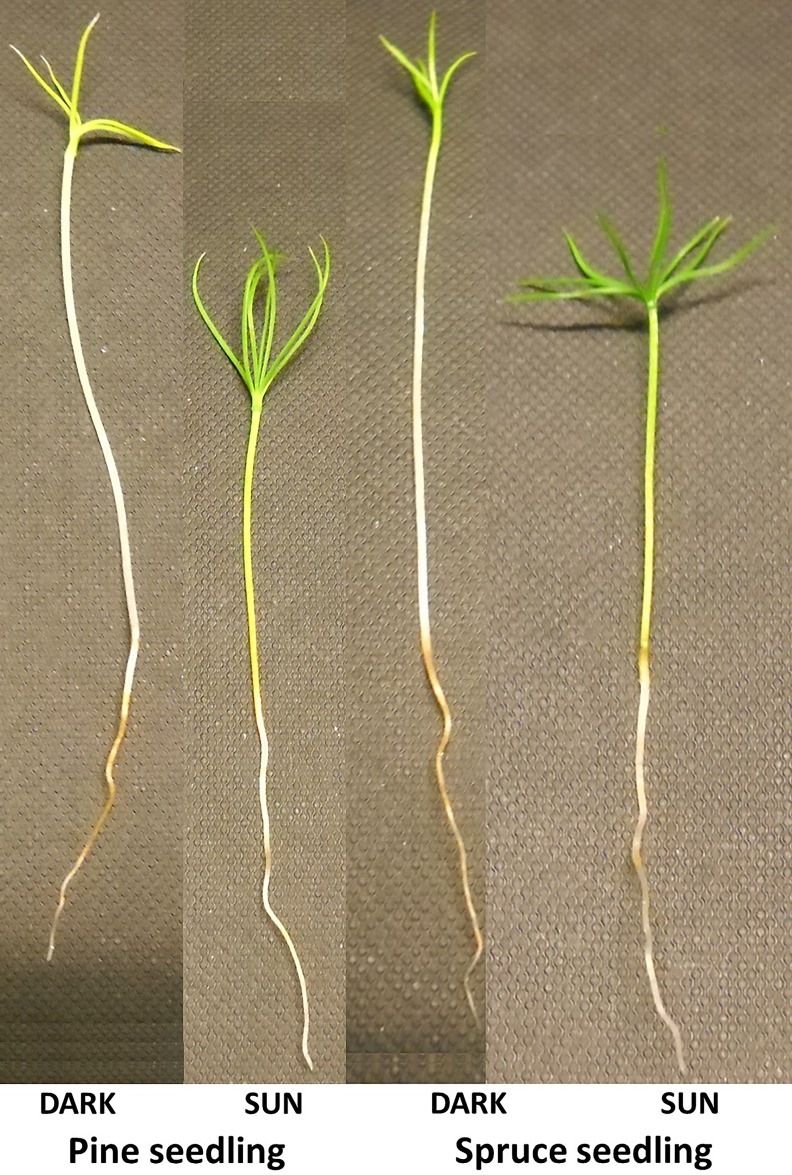
Scots pine and Norway spruce seedlings grown under DARK and SUN–Opening of cotyledons and pale green color due to synthesis of chlorophyll, in seedlings under DARK.

**Fig 2:**
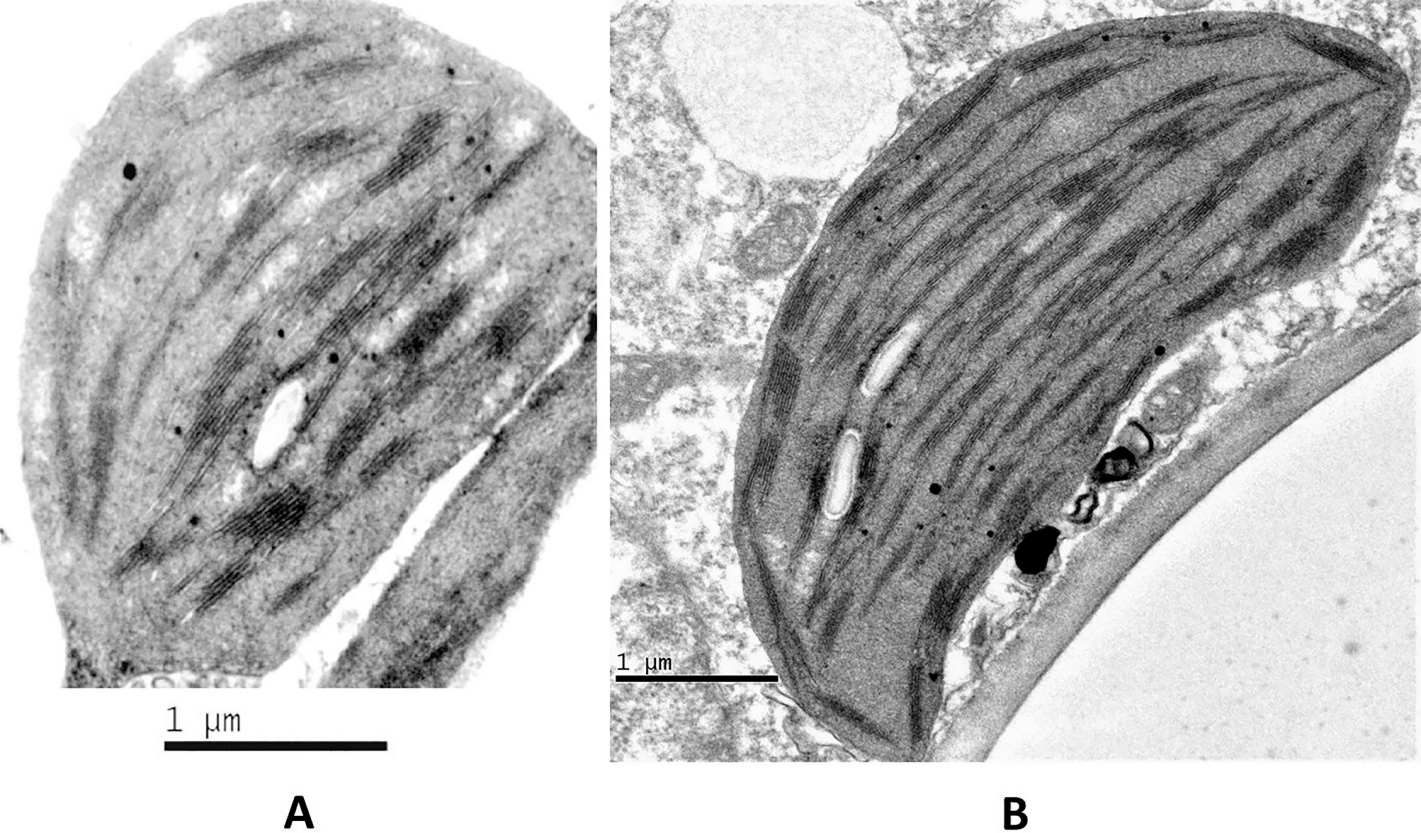
Transmission electron micrograph of chloroplast in Scots pine. (A) Formation of thylakoids in under DARK (B) Developed chloroplast under SUN.

As reported earlier [10], we did not observe the formation of apical hook in Scots pine and the cotyledons fully opened under DARK in both conifer species (Fig 1). DARK grown seedlings of both conifer species showed a pale green color due to the synthesis of chlorophyll. Synthesis of chlorophyll in pines under dark is demonstrated earlier [15, 36, 37]. Transmission electron micrograph (TEM) of chloroplast in Scots pine showed formation of thylakoids under DARK (Fig 2).

### Differentially expressed genes (DEG)

In both species the genes were classified into two groups, genes with higher expression under DARK with reference to SUN (DARK>SUN) and genes with lower expression under DARK (DARK<SUN) with respect to SUN. An overview of the data, including differentially regulated genes, raw and post-QC read counts, and alignment rates is included in Tables A-E in S1 File. PCA graphs with the original data and saturated heatmaps are included in Figures A and B, respectively in S2 File.

**Fig 3:**
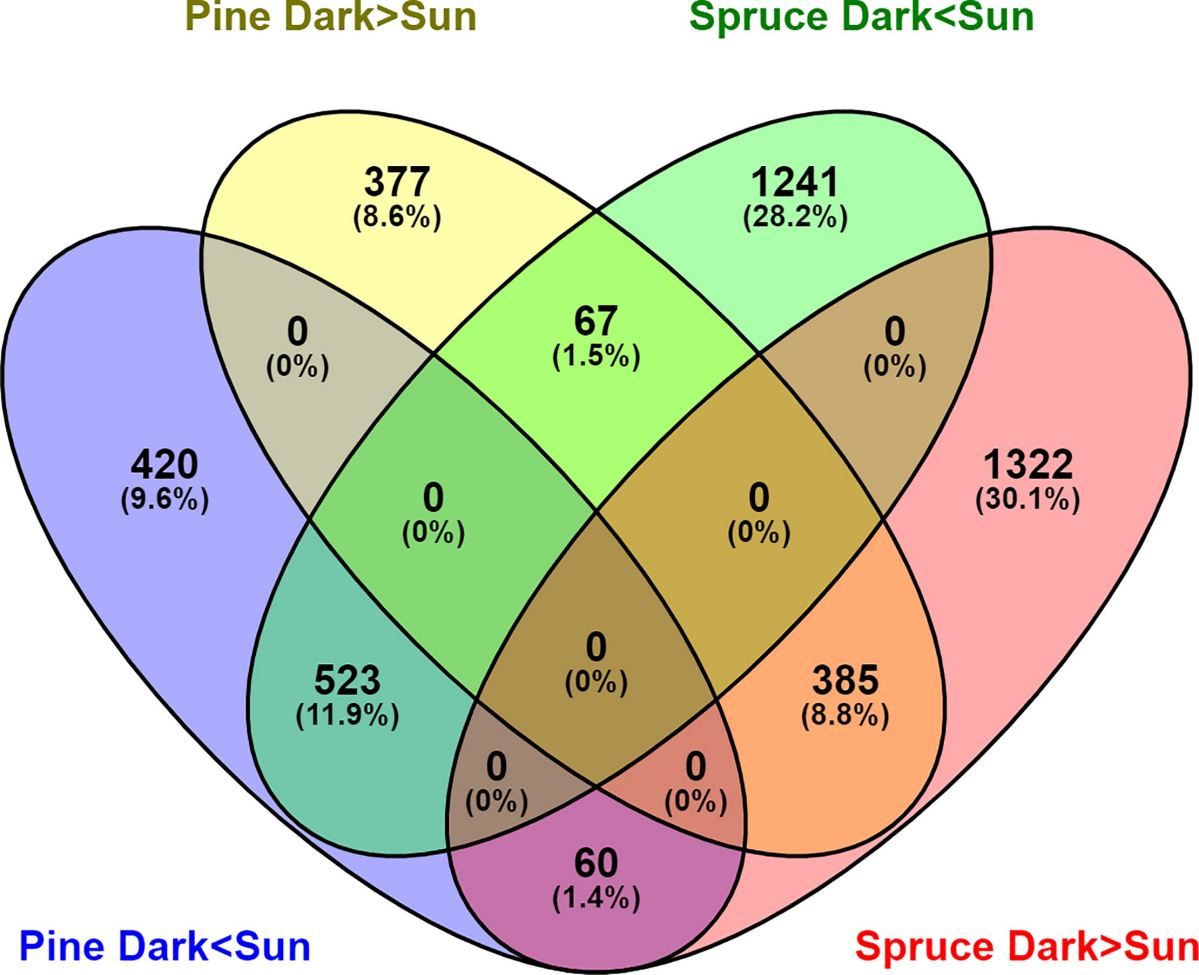
Venn diagram of the differentially expressed genes under DARK and SUN in Scots pine and Norway spruce.

In Scots pine, greater number of genes (*P-*value<0.01) were found with higher expression under SUN (1003) than the number of genes with higher expression under DARK (829), whilst in Norway spruce equal number of genes were detected with higher expression under SUN (1831) and DARK (1767) conditions (*P-*value>0.01). Venn diagram represents the number of common differentially expressed genes under DARK and SUN in Scots pine and Norway spruce (Fig 3).

**Fig 4:**
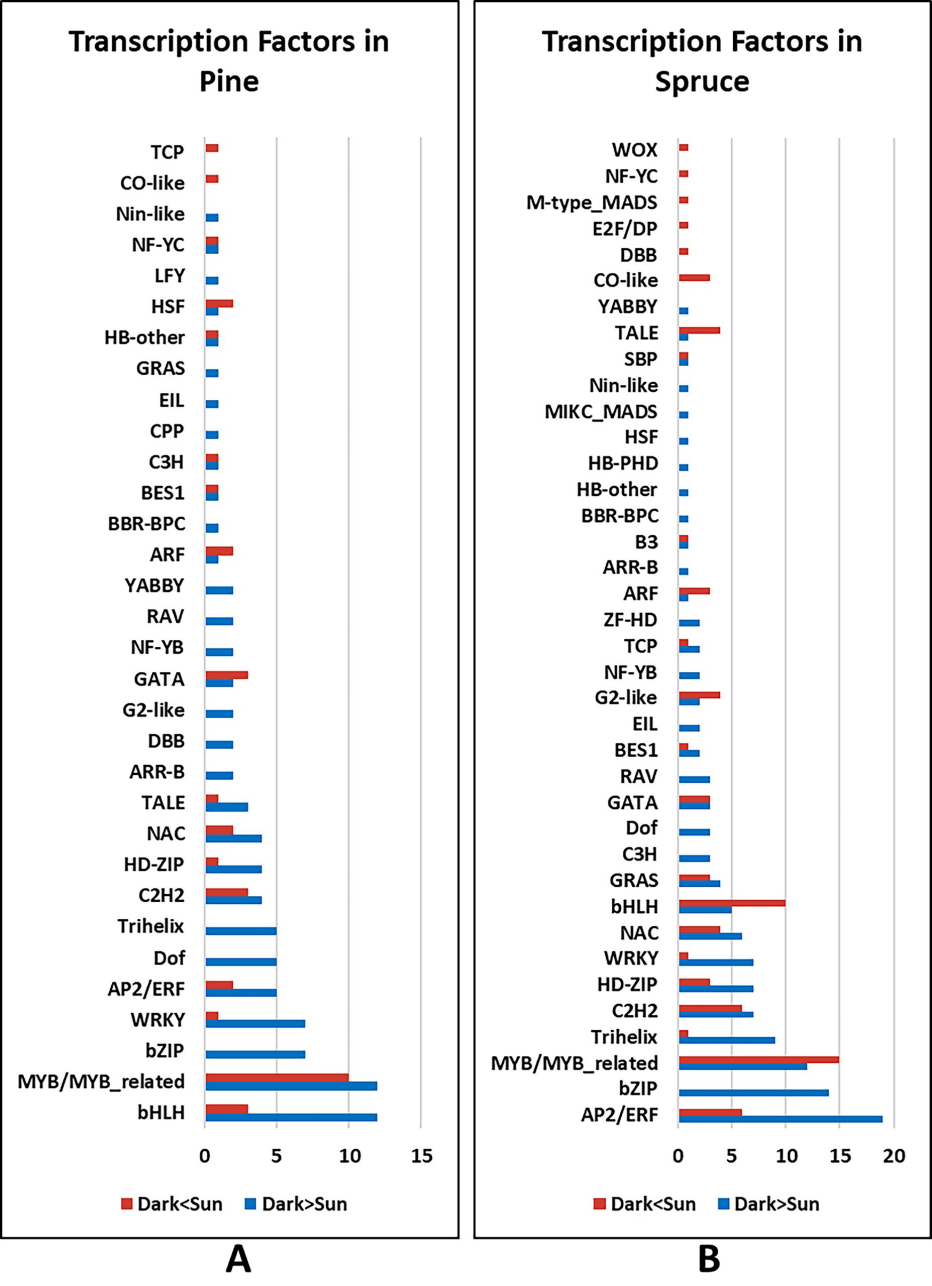
Transcription factors involved in etiolation and de-etiolation. (A) Scots pine and (B) Norway spruce.

A greater number of transcription factors (TF) (GO:0003700) were detected with higher expression under DARK as compared to the SUN condition in both the conifer species (*P-*value<0.01). Fig 4 represents the number of TFs associated with etiolation and de-etiolation in conifers, grouped according to the TF family using PlantTFDB 4.0 [38]. bHLH and MYB/MYB-related TF families are the most represented in Scots pine, while TFs from AP2/ERF, bZIP and MYB/MYB-related categories are most abundant in Norway Spruce. *HY5*, the key transcription factor involved in the process of photomorphogenesis showed higher expression under DARK in both conifer species. Expression of other TFs associated with etiolation/de-etiolation was found to be higher under DARK in both conifers (e.g. *Phytochrome interacting factor 3* (*PIF3*) and *TEMPRANILLO 1* (*TEM1*)). Table 1 represents the other candidate genes associated with photomorphogenesis in conifers, with defined functions in angiosperms (*A*. *thaliana*).

**Table 1:** Candidate genes associated with photomorphogenesis in conifers, with defined functions in *A*. *thaliana*.

Gene	Expression in conifers	Expression in *A*. *thaliana*	Defined function in *A*. *thaliana*	Reference
*COP1-interacting protein 8* (*CIP8*)	Higher expression under dark in spruce	Expressed in both light- and dark-grown seedlings	Interacts with COP1 and promotes degradation of COP1 targets e.g. HY5	[39]
*COP1 SUPPRESSOR 1 (CSU1)*	Higher expression under dark in spruce	Co-localizes with COP1 in nuclear speckles in dark	Targets COP1 for ubiquitination and degradation	[40]
*COP9-SIGNALOSOME 5B* (*CSN5B*)	Lower expression under DARK in both species	Higher expression during initial stage of seed germination	*COP/DET/FUS* encode proteins that suppress photomorphogenesis	[41]
*COP11/FUSCA6/CSN1*	Lower expression under DARK in Norway spruce	Higher expression during initial stage of seed germination	*COP/DET/FUS* encode proteins that suppress photomorphogenesis	[41]
*DE-ETIOLATED 1 (DET1)*	Lower expression under DARK in Scots pine	Expressed in both light- and dark-grown seedlings	*DET1-3* genes are essential for maintenance of skotomorphogenesis	[42–45]
*SPA1-RELATED 3 (SPA3)*	Lower expression under DARK in Scots pine	Higher expression under light	SPA1-SPA4 suppress photomorphogenesis in dark	[46]
*SPA1*, *SPA4*	Lower expression under DARK in Norway spruce	Higher expression under light	SPA1-SPA4 suppress photomorphogenesis in dark	[46]
*ANTHOCYANINLESS 2* (*ANL2*)	Higher expression under dark in both species	Anthocyanin synthesis is light depended	Involved in the accumulation of anthocyanin	[47, 48]
*CRYPTOCHROME1* (*CRY1*)	Higher expression under dark in Scots pine	Expression is not regulated by light	CRY1-SPA1 interaction plays role in COP1-dependent degradation of HY5	[49]
*PHYTOCHROME A (PHYA)*	Higher expression under DARK in Scots pine	Higher expression under dark	*PHYA* and *PHYB* interact to control de-etiolation	[50–53]
*PHYTOCHROME B (PHYB)*	Higher expression under SUN in Scots pine and higher under DARK in Norway spruce	Expressed in both light- and dark-grown seedlings	*PHYA* and *PHYB* interact to control de-etiolation	[52, 53]

### GO annotations

GO pie charts of molecular function (Figure C in S2 File) revealed that nucleic acid binding and transcription factor activity was higher under DARK as compared to SUN, while transporter activity increased under SUN in both species. With reference to biological process (Figure D in S2 File,), electron transport or energy pathways represented higher percentage while DNA dependent transcription was lower under SUN, in both the species. GO pie charts of cell component (Figure E in S2 File) showed that chloroplast and plastid represented higher percentage under SUN, while nucleus representation was found to be higher under DARK in both species.

### Metabolic pathway maps

The metabolic pathway maps (Figures A-L in S3 File) were constructed using MapMan 3.5.1R2 [54] with the DEGs associated with etiolation/de-etiolation in Scots pine and Norway spruce. Genes involved in cell-wall modification pathways (Figures A and G in S3 File) were found to be highly expressed in association to etiolation, while cellulose synthesis pathways were found to be more active in association to de-etiolation in conifers. Pathways involved in light reaction were up-regulated under SUN condition as expected. Regulation overview pathway indicated more number of TFs with higher expression under DARK in both conifer species. Equal number of genes were involved in the pathways related to development in DARK and SUN (Figures D and J in S3 File).

## Discussion

### Transcription factors

HY5 is a transcription factor which promotes expression of light-inducible genes leading to photomorphogenesis [55]. Expression of *HY5* was higher under DARK as compared to SUN in both species, perhaps due to negative feed-back loop. PIF3 is a transcription factor which triggers expression of skotomorphogenic genes in dark. PIFs degrade rapidly upon exposure to light through the process controlled by the light-activated phytochromes [56]. In the present study, *PIF3* showed higher expression under DARK as compared to the SUN condition, in both the conifers. Thus, *PIF3* expression appears to be conserved in the conifers when compared with angiosperms [1, 2]. *TEM1* is a transcription factor which plays key role in repressing flowering [57]. *TEM1* expression was found with higher expression under dark in both conifers. Suppression of flowering under DARK seems to be conserved in conifers; the floral induction is postponed until the plant has attained the specific growth stage [57].

### COP/DET/FUS associated with etiolation

COP1 represses photomorphogenesis and activates etiolation under dark by mediating ubiquitination and subsequent proteasomal degradation of light-induced transcription factors such as HY5; COP1 is critical for maintaining skotomorphogenesis namely hypocotyl elongation and closed cotyledons. Differential expression of *COP1* was not detected in both conifers, while *HY5* showed higher expression under DARK in both species. COP1-interacting protein 8 (*CIP8*) was detected with higher expression under dark in Norway spruce. CIP8 encodes RING-H2 protein that interacts with COP1 [39] and promotes ubiquitination, participating in the proteasome-mediated degradation of COP1 targets e.g. HY5. *COP9-SIGNALOSOME 5B* (*CSN5B*) was found with lower expression under DARK in both species. Additionally, *COP11/FUSCA6* showed lower expression under DARK in Norway spruce. Only one member from *COP/DET/FUS* was detected with higher expression under DARK—*COP12/FUSCA12* in Scots pine. *COP1 SUPPRESSOR 1* (*CSU1*) encodes RING-finger E3 ubiquitin ligase which plays a key role in maintaining COP1 homeostasis and targets COP1 for ubiquitination and degradation in dark-grown seedlings [40]. Higher expression of *CSU1* was detected under DARK in Norway spruce, suggesting degradation of COP1 by CSU1. *DET1-3* genes are essential for maintenance of skotomorphogenesis [43–45]; lower expression of *DET1* was found in Scots pine under DARK.

SPA protein family consists of at least four members—SPA1-SPA4, which function not only in suppressing photomorphogenesis in dark- and light-grown seedlings, but also regulate elongation growth in adult plants [46]. COP1-SPA1 interaction along with PHYTOCHROME A (PHYA) signaling, plays a critical role in degradation of HY5 leading to suppression of photomorphogenesis in dark [58]. Lower expression of *SPA3* was detected under DARK in Scots pine, while lower expression of *SPA1* and *SPA4* was detected under DARK in Norway spruce.

Skotomorphogenesis in Scots pine and Norway spruce is accompanied by synthesis of chlorophyll and opening of the cotyledons (Fig 1) which is not the characteristic feature of skotomorphogenesis in angiosperms (although the length of cotyledons is less in DARK as compared to SUN in conifers). As conifers differ from angiosperms in this aspect, the mechanism behind regulation and mode of action of *COP/DET/FUS* may follow an alternative pathway with reference to its role in the maintenance of etiolation in dark. Besides, COP/DET/FUS proteins also play key roles in many other biological processes such as growth and development [3]; for example DET1 is involved in the circadian rhythm [59] and COP1 controls circadian function and repression of photoperiodic flowering [60].

### Photoreceptors

CRYPTOCHROMEs (CRY) perceive the blue wavelength [61] and, mediate light regulation of cell elongation and flowering. *CRY1* expression was found to be higher under DARK in Scots pine while in Norway spruce, although the expression of *CRY1* was also significantly higher under DARK (*P-*value<0.01), the log_2_ fold change was only 0.47 (data not shown). *CRYPTOCHROME-INTERACTING BASIC-HELIX-LOOP-HELIX 5* (*CIB5*) was detected with higher expression under DARK in both conifers, *CIB5* interacts with *CRY2* and regulates flowering time [62]. CRY1-SPA1 interaction suppresses the COP1-SPA1 leading to suppression of COP1-dependent degradation of the transcription factor HY5, but CRY1-SPA1 interaction requires blue light [63]. Further, CRY1 and CRY2 negatively regulates COP1 in the presence of blue light through direct CRY-COP1 interaction [64]. However, conserved Glycine (CRY1: G380, CRY2: G377) in CRYs plays a critical role in regulating its photoreceptor activity [65]. Substitution of CRY1 (G380R) inhibits the nuclear accumulation of COP1 in darkness and displays a constitutively photomorphogenic phenotype [65]. The authors [65] concluded that CRY1 (G380R) might constitutively phenocopy the photo-activated CRY1 in darkness and thus constitutively mediate *CRY1* signaling. We propose that the conifer CRY sequences may differ from the angiosperms at a particular amino acid position (but position is conserved in conifers), due to which they can inhibit COP1 accumulation under DARK, which in turn may additionally suppress the degradation of HY5 under DARK leading to partial expression of photomorphogenic genes (S4 File).

PHYTOCHROMEs (PHY) perceive the R and the FR light [66]. PHYA and PHYB interact and also act in a redundant manner to control de-etiolation and flowering [67, 68]. Expression of *PHYA/N* was higher under DARK in Scots pine while *PHYB/P* was detected with higher expression under DARK in Norway spruce.

### Pigment synthesis

Conifer chloroplast genome possess additional light-independent enzyme, dark-operative DPOR, which is responsible for the enzymatic reduction of protochlorophyllide to chlorophyllide, the key step in chlorophyll biosynthesis [13]. Unlike nuclear encoded mRNA, most of the chloroplast mRNAs are non-polyadenylated at the 3′-end. We followed an RNA-Seq protocol that selects for poly-adenylated transcripts, therefore in the present study, differential regulation of *DPOR* or genes encoding its subunits (*chlL*, *chlN*, and *chlB*) were not detected in the conifer species, but Fig 1 shows the pale green color of the DARK grown seedlings of both conifer species. Electron micrograph of the chloroplast in the DARK grown seedlings shows formation of thylakoids in Scots pine (Fig 2). Synthesis of chlorophyll in dark by pine and spruce is also reported by earlier investigations [14, 15, 37]. Higher expression of *ANTHOCYANINLESS 2* (*ANL2*), a transcription factor which is involved in the accumulation of anthocyanin and root development [48], was detected under DARK in both species.

### Proposed mechanism involved in etiolation/de-etiolation in conifers

**Fig 5:**
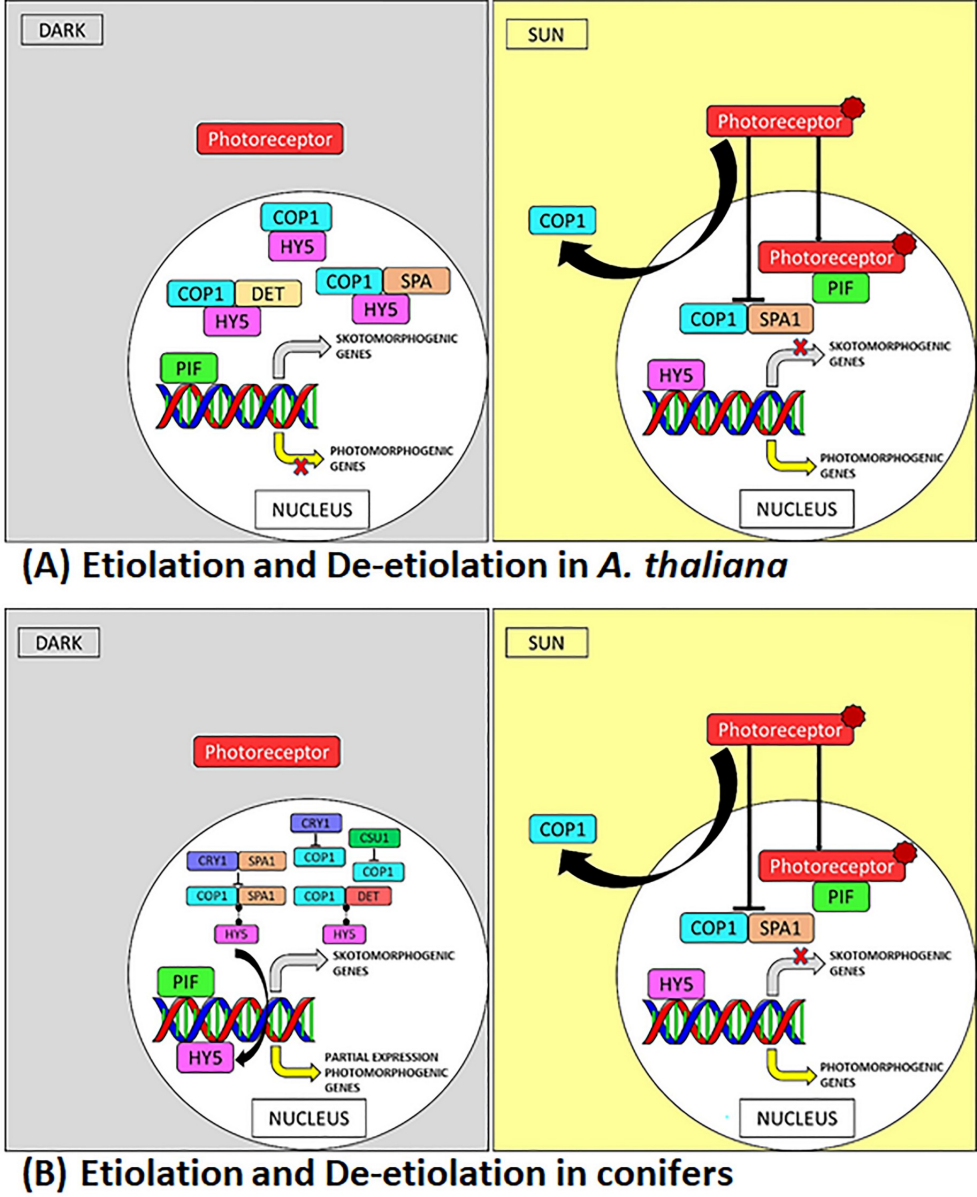
Comparison of the process of etiolation and de-etiolation in *A*. *thaliana* and conifers. (A) Etiolation and de-etiolation in *A*. *thaliana* (B) Proposed mechanism for etiolation and de-etiolation in conifers.

In *A*. *thaliana*, HY5 is degraded under dark through COP1 activity, thus suppressing the expression of photomorphogenic genes (Fig 5A). We propose a putative mechanism involved in etiolation/de-etiolation from the RNA-Seq data analysis and the DARK phenotypes in the two studied conifer species (Fig 5B). In conifers, the expression of *SPA* (*SPA1*, *SPA3*, *SPA4*), *DET* (*DET 1*, *DET 3*) and *CSN5B* was lower under DARK, therefore COP1-SPA and COP1-DET mediated HY5 degradation might be low. Besides, in Norway spruce, COP1 might be subjected to ubiquitination and degradation by CSU1 under DARK (higher expression of *CSU1* under DARK). Additionally, higher expression of *CRY1* in DARK which is known to inhibit the nuclear accumulation of COP1 in the dark, may also contribute to lower the COP1-mediated degradation of HY5. CRY1-SPA1 interaction may also suppress the COP1-SPA1 complex which in turn degrades HY5. Although majority of HY5 might be subjected to degradation by COP1 alone, but due to the higher expression of *HY5* under DARK in both the conifer species coupled with proposed theories behind lower degradation or lack of complete degradation of HY5, some quantity of HY5 may remain intact that binds to DNA and promotes partial expression of photomorphogenic genes under DARK in conifers leading to display of some photomorphogenic characters in the DARK such as absence of apical hook, opening of cotyledons, development of thylakoids and synthesis of chlorophyll; also stimulating expression of few genes related to biosynthesis of anthocyanin. Conifers can synthesize chlorophyll in the dark due to the presence of *chlL*, *chlN*, and *chlB* in the chloroplast genome. Molecular mechanisms for regulation of these genes is not understood, it is possible that HY5 controls the expression of these genes in the dark leading to synthesis of chlorophyll. The current work includes an RNA-Seq data analysis with sufficient statistical evidence and a strong supportive phenotypic validation associated with the expression of the candidate genes that explain the proposed mechanism involved in etiolation/de-etiolation in conifers e.g. absence of apical hook, opening of cotyledons (Fig 1), development of thylakoids (Fig 2), synthesis of chlorophyll and anthocyanin accumulation (higher expression of few genes from flavonoid synthesis pathway under DARK) in the DARK grown conifer seedlings which is similar to some of the features of photomorphogenic seedling phenotype in dark, displayed by *A*. *thaliana* mutants of the same candidate genes (*cop*/*det*/*fus*) [69, 70]. Moreover, conifer seedlings under DARK are not as elongated as *A*. *thaliana* seedlings under DARK. In other words, the ratio of the hypocotyl lengths of the conifer seedlings grown under DARK and SUN is lower than the ratio in *A*. *thaliana* seedlings grown under dark and sun conditions [71]. This shows that there is some inhibition of the hypocotyl elongation under DARK in conifers which is similar to the feature displayed by *cop1* mutants in *A*. *thaliana*, where *cop1* mutants display a short hypocotyl under dark [72]. Lack of, or diminished, COP1 activity with reference to HY5 degradation under DARK in the conifers could be linked to the display of the associated phenotype similar to *cop1* mutant in *A*. *thaliana* that accumulates HY5 in the dark [55]. *PIF* expression was detected to be higher under DARK and in absence of activated phytochromes which target the PIF, PIF promotes expression of skotomorphogenic genes under DARK.

We also propose that similar to angiosperms (Fig 5A), HY5 accumulates in the presence of light in SUN conditions in conifers (Fig 5B) due to the nuclear exclusion of COP1 through the mechanism involving photoreceptors and promotes expression genes of specific to photomorphogenesis. Light-activated phytochromes interact with PIFs, thereby inducing rapid degradation of PIFs leading to photomorphogenesis [56, 73]. Thus, the process of photomorphogenesis/de-etiolation appears to be conserved in conifers.

The mechanism proposed for the process of etiolation in conifers is solely based on gene expression analyses and further research is required to understand the process at the protein level e.g. to determine how COP1 remains partially inactive under DARK, albeit our expression results led us to propose a few mechanisms to follow up. From the CRY1, HY5 and PIF3 alignments it is clear that conifers protein sequences are different from angiosperms, and may acquire different conformation, therefore behave differently. It is equally important to understand how *HY5* selectively binds to specific genes leading to partial expression of photomorphogenic genes, although its expression is higher under DARK.

## Conclusions

The gene expression analysis and the associated phenotype suggests that the process of de-etiolation is conserved in conifers, but conifers are different from angiosperms with reference to etiolation. Etiolated conifer seedlings exhibit some of the typical characteristics of de-etiolated phenotype of the angiosperm species. Moreover, DARK grown conifer seedlings display absence of apical hook, extended/open cotyledons and development of chloroplast; a phenotype which is similar to the dark grown *A*. *thaliana* mutants of *cop/det/fus* genes, the key genes that play a central role in maintaining skotomorphogenesis [70]. Differential expression of these genes in conifer etiolated and de-etiolated seedlings, and the associated phenotype suggest a role of these genes in the process of etiolation and de-etiolation in conifers. Conifers also synthesize chlorophyll in DARK, which is primarily a light-dependent activity and the underlying regulatory mechanism needs further research. It is also possible that the conifer COP1 possesses different biochemical properties that functions through an alternative molecular pathway that warrants further investigation.

## Supporting information

S1 File Supplementary tables. **Table A—PineDark<Sun**—Genes with significant higher expression under SUN in Scots pine.
**Table B—PineDark>Sun**—Genes with significant higher expression under DARK in Scots pine.
**Table C—SpruceDark<Sun**—Genes with significant higher expression under SUN in Norway spruce.
**Table D—SpruceDark>Sun**—Genes with significant higher expression under DARK in Norway spruce.
**Table E**—Raw and post-QC read counts, and alignment rates
(XLSX)

S2 File Supplementary figures. **Figure A** PCA based on the entire transcriptome
(a) Scots pine
(b) Norway spruce
**Figure B** Heatmaps for the entire transcriptome
(a) Scots pine
(b) Norway spruce
**Figure C** GO Molecular Function: Functional categorization by annotation—pie charts for Scots pine and Norway spruce. Percentage denotes Number of annotations to terms in the GOslim category * 100 / Number of total annotations to terms in the ontology. Number in the parenthesis denotes number of genes in the respective functional category.
**Figure D** GO Biological Process: Functional categorization by annotation—pie charts for Scots pine and Norway spruce. Percentage denotes Number of annotations to terms in the GOslim category * 100 / Number of total annotations to terms in the ontology. Number in the parenthesis denotes number of genes in the respective functional category.
**Figure E** GO Cellular Component: Functional categorization by annotation—pie charts for Scots pine and Norway spruce. Percentage denotes Number of annotations to terms in the GOslim category * 100 / Number of total annotations to terms in the ontology. Number in the parenthesis denotes number of genes in the respective functional category.
(PDF)

S3 File Metabolic pathway maps. The metabolic pathway maps constructed using MapMan 3.5.1R2 with the DEGs associated with etiolation/de-etiolation in Scots pine and Norway Spruce.
**Figure A** Metabolism overview in Scots pine
**Figure B** Regulation overview in Scots pine
**Figure C** Cell function overview in Scots pine
**Figure D** Cellular response overview in Scots pine
**Figure E** Secondary metabolism in Scots pine
**Figure F** Transcription in Scots pine
**Figure G** Metabolism overview in Norway Spruce
**Figure H** Regulation overview in Norway Spruce
**Figure I** Cell function overview in Norway Spruce
**Figure J** Cellular response overview in Norway Spruce
**Figure K** Secondary metabolism in Norway Spruce
**Figure L** Transcription in Norway Spruce.
(PDF)

S4 File Protein alignments of CRY1, PIF3 and HY5. Protein alignments of CRY1, PIF3 and HY5 from angiosperm and gymnosperm species.
(PDF)

## References

[1] Leivar P, Monte E, Oka Y, Liu T, Carle C, Castillon A, et al Multiple phytochrome-interacting bHLH transcription factors repress premature seedling photomorphogenesis in darkness. Current Biology. 2008;18(23):1815–23. WOS:000261721100021. 10.1016/j.cub.2008.10.058 19062289 PMC2651225

[2] Leivar P, Monte E. PIFs: Systems integrators in plant development. Plant Cell. 2014;26(1):56–78. WOS:000332223200008. 10.1105/tpc.113.120857 24481072 PMC3963594

[3] Lau OS, Deng XW. The photomorphogenic repressors COP1 and DET1: 20 years later. Trends Plant Sci. 2012;17(10):584–93. Epub 2012/06/19. 10.1016/j.tplants.2012.05.004 . 22705257

[4] Yanagawa Y, Sullivan JA, Komatsu S, Gusmaroli G, Suzuki G, Yin JN, et al *Arabidopsis* COP10 forms a complex with DDB1 and DET1 in vivo and enhances the activity of ubiquitin conjugating enzymes. Gene Dev. 2004;18(17):2172–81. WOS:000223675200013. 10.1101/gad.1229504 15342494 PMC515294

[5] Wei N, Serino G, Deng XW. The COP9 signalosome: more than a protease. Trends Biochem Sci. 2008;33(12):592–600. WOS:000261836700005. 10.1016/j.tibs.2008.09.004 18926707

[6] Zhu DM, Maier A, Lee JH, Laubinger S, Saijo Y, Wang H, et al Biochemical characterization of *Arabidopsis* complexes containing CONSTITUTIVELY PHOTOMORPHOGENIC1 and SUPPRESSOR OF PHYA proteins in light control of plant development. Plant Cell. 2008;20(9):2307–23. WOS:000260425900003. 10.1105/tpc.107.056580 18812498 PMC2570740

[7] Gangappa SN, Botto JF. The multifaceted roles of HY5 in plant growth and development. Mol Plant. 2016;9(10):1353–65. Epub 2016/07/21. 10.1016/j.molp.2016.07.002 . 27435853

[8] Ang LH, Chattopadhyay S, Wei N, Oyama T, Okada K, Batschauer A, et al Molecular interaction between COP1 and HY5 defines a regulatory switch for light control of *Arabidopsis* development. Mol Cell. 1998;1(2):213–22. 9659918 10.1016/s1097-2765(00)80022-2

[9] Yi CL, Deng XW. COP1—from plant photomorphogenesis to mammalian tumorigenesis. Trends Cell Biol. 2005;15(11):618–25. WOS:000233732100009. 10.1016/j.tcb.2005.09.007 16198569

[10] Hřib J, Lánícková B. Circumnutation oscillations of the hypocotyl and hypocotyl hook formation: A comparison of Norway spruce (*Picea abies* (L.)Karst.) and Scots pine (*Pinus silvestris* L.) seedlings. Biologia Plantarum. 1986;28(180).

[11] Spurný M. Elongation and circumnutation oscillations of hypocotyl of pine seedlings (*Pinus silvestris* L.). Biologia Plantarum. 1975;17(1):43–9.

[12] Schoefs B, Bertrand M. Chlorophyll biosynthesis In: Pessarakli M, editor. Handbook of Photosynthesis. Second ed. New York: Marcel Dekker; 1997 p. pp 49–71.

[13] Armstrong GA. Greening in the dark: light-independent chlorophyll biosynthesis from anoxygenic photosynthetic bacteria to gymnosperms. J Photoch Photobio B. 1998;43(2):87–100.

[14] Stolarik T, Hedtke B, Santrucek J, Ilik P, Grimm B, Pavlovic A. Transcriptional and post-translational control of chlorophyll biosynthesis by dark-operative protochlorophyllide oxidoreductase in Norway spruce. Photosynthesis Research. 2017;132(2):165–79. WOS:000399255800007. 10.1007/s11120-017-0354-2 28229362

[15] Schoefs B, Franck F. Chlorophyll synthesis in dark-grown pine primary needles. Plant Physiol. 1998;118(4):1159–68. WOS:000077477600007. 10.1104/pp.118.4.1159 9847090 PMC34732

[16] Franklin KA, Whitelam GC. Phytochrome a function in red light sensing. Plant Signal Behav. 2007;2(5):383–5. Epub 2007/09/01. 10.4161/psb.2.5.4261 19704607 PMC2634220

[17] Li J, Li G, Wang H, Wang Deng X. Phytochrome signaling mechanisms. Arabidopsis Book. 2011;9:e0148 Epub 2012/02/04. 10.1199/tab.0148 22303272 PMC3268501

[18] Ranade SS, García-Gil MR. Ecotypic variation in response to light spectra in Scots pine (*Pinus sylvestris* L.). Tree Physiology. 2013;33(2):195–201. WOS:000315435600008. 10.1093/treephys/tps131 23392595

[19] Razzak A, Ranade SS, Strand A, Garcia-Gil MR. Differential response of Scots pine seedlings to variable intensity and ratio of red and far-red light. Plant Cell and Environment. 2017;40(8):1332–40. 10.1111/pce.12921 . 28108999

[20] Clapham DH, Dormling I, Ekberg I, Eriksson G, Qamaruddin M, Vince-Prue D. Latitudinal cline of requirement for far-red light for the photoperiodic control of budset and extension growth in *Picea abies* (Norway spruce). Physiologia Plantarum. 1998;102(1):71–8. ISI:000072529800010. 10.1034/j.1399-3054.1998.1020110.x 35359127

[21] Clapham DH, Ekberg I, Eriksson G, Norell L, Vince-Prue D. Requirement for far-red light to maintain secondary needle extension growth in northern but not southern populations of *Pinus sylvestris* (Scots pine). Physiologia Plantarum. 2002;114(2):207–12. WOS:000174551100006. 11903967 10.1034/j.1399-3054.2002.1140206.x

[22] Spurr AR. A low-viscosity epoxy resin embedding medium for electron microscopy. J Ultra Mol Struct R. 1969;26(1–2):31–&. 10.1016/S0022-5320(69)90033-1 WOS:A1969D012100004. 4887011

[23] Lundin S, Stranneheim H, Pettersson E, Klevebring D, Lundeberg J. Increased throughput by parallelization of library preparation for massive sequencing. Plos One. 2010;5(4):e10029 10.1371/journal.pone.0010029 20386591 PMC2850305

[24] Borgstrom E, Lundin S, Lundeberg J. Large scale library generation for high throughput sequencing. Plos One. 2011;6(4):e19119 10.1371/journal.pone.0019119 21589638 PMC3083417

[25] Kopylova E, Noe L, Touzet H. SortMeRNA: fast and accurate filtering of ribosomal RNAs in metatranscriptomic data. Bioinformatics. 2012;28(24):3211–7. 10.1093/bioinformatics/bts611 . 23071270

[26] Bolger AM, Lohse M, Usadel B. Trimmomatic: a flexible trimmer for Illumina sequence data. Bioinformatics. 2014;30(15):2114–20. 10.1093/bioinformatics/btu170 24695404 PMC4103590

[27] Sundell D, Mannapperuma C, Netotea S, Delhomme N, Lin YC, Sjodin A, et al The Plant Genome Integrative Explorer Resource: PlantGenIE.org. New Phytologist. 2015;208(4):1149–56. 10.1111/nph.13557 . 26192091

[28] Dobin A, Davis CA, Schlesinger F, Drenkow J, Zaleski C, Jha S, et al STAR: ultrafast universal RNA-seq aligner. Bioinformatics. 2013;29(1):15–21. 10.1093/bioinformatics/bts635 23104886 PMC3530905

[29] Anders S, Pyl PT, Huber W. HTSeq—a Python framework to work with high-throughput sequencing data. Bioinformatics. 2015;31(2):166–9. 10.1093/bioinformatics/btu638 25260700 PMC4287950

[30] Zimin A, Stevens KA, Crepeau M, Holtz-Morris A, Koriabine M, Marcais G, et al Sequencing and assembly of the 22-Gb Loblolly pine genome. Genetics. 2014;196(3):875–90. WOS:000333905500023. 10.1534/genetics.113.159715 24653210 PMC3948813

[31] R Development Core Team R. R: A language and environment for statistical computing R Foundation for Statistical Computing, Vienna, Austria, ISB. 2015.

[32] Gentleman RC, Carey VJ, Bates DM, Bolstad B, Dettling M, Dudoit S, et al Bioconductor: open software development for computational biology and bioinformatics. Genome Biology. 2004;5(10):R80 10.1186/gb-2004-5-10-r80 15461798 PMC545600

[33] Love MI, Huber W, Anders S. Moderated estimation of fold change and dispersion for RNA-seq data with DESeq2. Genome Biology. 2014;15(12):550 10.1186/s13059-014-0550-8 25516281 PMC4302049

[34] Sundell D, Mannapperuma C, Netotea S, Delhomme N, Lin YC, Sjodin A, et al The Plant Genome Integrative Explorer Resource: PlantGenIE.org. New Phytol. 2015;208(4):1149–56. Epub 2015/07/21. 10.1111/nph.13557 . 26192091

[35] Nystedt B, Street NR, Wetterbom A, Zuccolo A, Lin YC, Scofield DG, et al The Norway spruce genome sequence and conifer genome evolution. Nature. 2013;497(7451):579–84. WOS:000319556100035. 10.1038/nature12211 23698360

[36] Bogdanov M. Chlorophyll formation in dark. 1. Chlorophyll in pine seedlings. Physiologia Plantarum. 1973;29(1):17–8. WOS:A1973Q786100004.

[37] Garcia-Gutierrez A, Dubois F, Canton FR, Gallardo F, RS Sangwan, Canovas FM. Two different modes of early development and nitrogen assimilation in gymnosperm seedlings. Plant J. 1998;13(2):187–99. WOS:000072088600004.

[38] Jin J, Tian F, Yang DC, Meng YQ, Kong L, Luo J, et al PlantTFDB 4.0: toward a central hub for transcription factors and regulatory interactions in plants. Nucleic Acids Res. 2017;45(D1):D1040–D5. Epub 2016/12/08. 10.1093/nar/gkw982 27924042 PMC5210657

[39] Torii KU, Stoop-Myer CD, Okamoto H, Coleman JE, Matsui M, Deng XW. The RING finger motif of photomorphogenic repressor COP1 specifically interacts with the RING-H2 motif of a novel *Arabidopsis* protein. J Biol Chem. 1999;274(39):27674–81. Epub 1999/09/17. 10.1074/jbc.274.39.27674 . 10488108

[40] Xu D, Lin F, Jiang Y, Huang X, Li J, Ling J, et al The RING-finger E3 ubiquitin ligase COP1 SUPPRESSOR1 negatively regulates COP1 abundance in maintaining COP1 homeostasis in dark-Grown *Arabidopsis* seedlings. Plant Cell. 2014;26(5):1981–91. Epub 2014/05/20. 10.1105/tpc.114.124024 24838976 PMC4079363

[41] Klepikova AV, Kasianov AS, Gerasimov ES, Logacheva MD, Penin AA. A high resolution map of the *Arabidopsis thaliana* developmental transcriptome based on RNA-seq profiling. Plant J. 2016;88(6):1058–70. WOS:000393126200012. 10.1111/tpj.13312 27549386

[42] Anderson SL, Kay SA. Phototransduction and circadian clock pathways regulating gene transcription in higher plants. Adv Genet. 1997;35:1–34. 10.1016/S0065-2660(08)60446-0 WOS:A1997BH43Z00001. 9348644

[43] Schumacher K, Vafeados D, McCarthy M, Sze H, Wilkins T, Chory J. The *Arabidopsis* det3 mutant reveals a central role for the vacuolar H(+)-ATPase in plant growth and development. Genes Dev. 1999;13(24):3259–70. Epub 2000/01/05. 10.1101/gad.13.24.3259 10617574 PMC317205

[44] Chory J, Nagpal P, Peto CA. Phenotypic and genetic analysis of det2, a new mutant that affects light-regulated seedling development in *Arabidopsis*. Plant Cell. 1991;3(5):445–59. Epub 1991/05/01. 10.1105/tpc.3.5.445 12324600 PMC160013

[45] Schroeder DF, Gahrtz M, Maxwell BB, Cook RK, Kan JM, Alonso JM, et al De-etiolated 1 and damaged DNA binding protein 1 interact to regulate *Arabidopsis* photomorphogenesis. Curr Biol. 2002;12(17):1462–72. Epub 2002/09/13. . 12225661 10.1016/s0960-9822(02)01106-5

[46] Fittinghoff K, Laubinger S, Nixdorf M, Fackendahl P, Baumgardt RL, Batschauer A, et al Functional and expression analysis of *Arabidopsis* SPA genes during seedling photomorphogenesis and adult growth. Plant J. 2006;47(4):577–90. WOS:000239373400007. 10.1111/j.1365-313X.2006.02812.x 16813571

[47] Mancinelli AL. Light-dependent anthocyanin synthesis: A model system for the study of plant photomorphogenesis. Bot Rev. 1985;51(1):107–57. 10.1007/Bf02861059 WOS:A1985ADG1900003.

[48] Kubo H, Peeters AJ, Aarts MG, Pereira A, Koornneef M. ANTHOCYANINLESS2, a homeobox gene affecting anthocyanin distribution and root development in *Arabidopsis*. Plant Cell. 1999;11(7):1217–26. Epub 1999/07/13. 10.1105/tpc.11.7.1217 10402424 PMC144283

[49] Batschauer A. Plant cryptochromes: Their genes, biochemistry, and physiological roles In: Briggs W, Spudich J, editors. Handbook of Photosensory Receptors. Weinheim, Germany: Wiley‐VCH; 2005 p. 211–46.

[50] Quail PH. Phytochrome—a light-activated molecular switch that regulates plant gene-expression. Annu Rev Genet. 1991;25:389–409. WOS:A1991GX17400016. 10.1146/annurev.ge.25.120191.002133 1812812

[51] Sharrock RA, Quail PH. Novel phytochrome sequences in *Arabidopsis thaliana*: structure, evolution, and differential expression of a plant regulatory photoreceptor family. Gene Dev. 1989;3(11):1745–57. WOS:A1989CK68800010. 10.1101/gad.3.11.1745 2606345

[52] Clack T, Mathews S, Sharrock RA. The phytochrome apoprotein family in *Arabidopsis* is encoded by five genes: the sequences and expression ofPHYD and PHYE. Plant Mol Biol. 1994;25(3):413–27. WOS:A1994PB08100009. 8049367 10.1007/BF00043870

[53] Su L, Hou P, Song MF, Zheng X, Guo L, Xiao Y, et al Synergistic and antagonistic action of phytochrome (Phy) A and PhyB during seedling de-Etiolation in *Arabidopsis thaliana*. Int J Mol Sci. 2015;16(6):12199–212. WOS:000357492800023. 10.3390/ijms160612199 26030677 PMC4490439

[54] Thimm O, Blasing O, Gibon Y, Nagel A, Meyer S, Kruger P, et al MAPMAN: a user-driven tool to display genomics data sets onto diagrams of metabolic pathways and other biological processes. Plant J. 2004;37(6):914–39. Epub 2004/03/05. . 14996223 10.1111/j.1365-313x.2004.02016.x

[55] Osterlund MT, Hardtke CS, Wei N, Deng XW. Targeted destabilization of HY5 during light-regulated development of *Arabidopsis*. Nature. 2000;405(6785):462–6. WOS:000087212000050. 10.1038/35013076 10839542

[56] Leivar P, Quail PH. PIFs: pivotal components in a cellular signaling hub. Trends Plant Sci. 2011;16(1):19–28. Epub 2010/09/14. 10.1016/j.tplants.2010.08.003 20833098 PMC3019249

[57] Osnato M, Castillejo C, Matias-Hernandez L, Pelaz S. TEMPRANILLO genes link photoperiod and gibberellin pathways to control flowering in *Arabidopsis*. Nat Commun. 2012;3:808 Epub 2012/05/03. 10.1038/ncomms1810 . 22549837

[58] Saijo Y, Sullivan JA, Wang H, Yang J, Shen Y, Rubio V, et al The COP1-SPA1 interaction defines a critical step in phytochrome A-mediated regulation of HY5 activity. Genes Dev. 2003;17(21):2642–7. Epub 2003/11/05. 10.1101/gad.1122903 14597662 PMC280614

[59] McClung CR. The photomorphogenic protein, DE-ETIOLATED 1, is a critical transcriptional corepressor in the central loop of the *Arabidopsis* circadian clock. Mol Cell. 2011;43(5):693–4. Epub 2011/09/03. 10.1016/j.molcel.2011.08.013 . 21884969

[60] Xu D, Zhu D, Deng XW. The role of COP1 in repression of photoperiodic flowering. F1000Res. 2016;5 Epub 2016/03/08. 10.12688/f1000research.7346.1 26949521 PMC4756798

[61] Christie JM, Briggs WR. Blue light sensing in higher plants. The Journal of biological chemistry. 2001;276(15):11457–60. Epub 2001/03/30. 10.1074/jbc.R100004200 . 11279226

[62] Liu HT, Yu XH, Li KW, Klejnot J, Yang HY, Lisiero D, et al Photoexcited CRY2 interacts with CIB1 to regulate transcription and floral initiation in *Arabidopsis*. Science. 2008;322(5907):1535–9. WOS:000261377400047. 10.1126/science.1163927 18988809

[63] Liu B, Zuo ZC, Liu HT, Liu XM, Lin CT. *Arabidopsis* cryptochrome 1 interacts with SPA1 to suppress COP1 activity in response to blue light. Gene Dev. 2011;25(10):1029–34. 10.1101/gad.2025011 21511871 PMC3093118

[64] Wang H, Ma LG, Li JM, Zhao HY, Deng XW. Direct interaction of *Arabidopsis* cryptochromes with COP1 in light control development. Science. 2001;294(5540):154–8. Epub 2001/08/18. 10.1126/science.1063630 . 11509693

[65] Gu NN, Zhang YC, Yang HQ. Substitution of a conserved glycine in the PHR domain of *Arabidopsis* cryptochrome 1 confers a constitutive light response. Mol Plant. 2012;5(1):85–97. Epub 2011/07/19. 10.1093/mp/ssr052 . 21765176

[66] Smith H. and Phytochromes light signal perception by plants—an emerging synthesis. Nature. 2000;407(6804):585–91. Epub 2000/10/18. 10.1038/35036500 . 11034200

[67] Takano M, Inagaki N, Xie X, Yuzurihara N, Hihara F, Ishizuka T, et al Distinct and cooperative functions of phytochromes A, B, and C in the control of deetiolation and flowering in rice. Plant Cell. 2005;17(12):3311–25. Epub 2005/11/10. 10.1105/tpc.105.035899 16278346 PMC1315371

[68] Weller JL, Beauchamp N, Kerckhoffs LH, Platten JD, Reid JB. Interaction of phytochromes A and B in the control of de-etiolation and flowering in pea. Plant J. 2001;26(3):283–94. Epub 2001/07/06. . 11439117 10.1046/j.1365-313x.2001.01027.x

[69] Deng XW, Caspar T, Quail PH. cop1: a regulatory locus involved in light-controlled development and gene expression in *Arabidopsis*. Genes Dev. 1991;5(7):1172–82. Epub 1991/07/01. 10.1101/gad.5.7.1172 . 2065972

[70] Hardtke CS, Deng XW. The cell biology of the COP/DET/FUS proteins. Regulating proteolysis in photomorphogenesis and beyond? Plant Physiol. 2000;124(4):1548–57. Epub 2000/12/15. 10.1104/pp.124.4.1548 11115873 PMC1539311

[71] Morelli G, Ruberti I. Shade avoidance responses. Driving auxin along lateral routes. Plant Physiol. 2000;122(3):621–6. Epub 2000/03/11. 10.1104/pp.122.3.621 10712524 PMC1539242

[72] McNellis TW, von Arnim AG, Araki T, Komeda Y, Misera S, Deng XW. Genetic and molecular analysis of an allelic series of cop1 mutants suggests functional roles for the multiple protein domains. Plant Cell. 1994;6(4):487–500. Epub 1994/04/01. 10.1105/tpc.6.4.487 8205001 PMC160452

[73] Castillon A, Shen H, Huq E. Phytochrome Interacting Factors: central players in phytochrome-mediated light signaling networks. Trends Plant Sci. 2007;12(11):514–21. Epub 2007/10/16. 10.1016/j.tplants.2007.10.001 . 17933576

