# Supplementary figures and images for: Global gene expression analysis in etiolated and de-etiolated seedlings in conifers
Source: PLoS One. 2019 Jul 5;14(7):e0219272. doi: 10.1371/journal.pone.0219272 (PMC6611632; doi:10.1371/journal.pone.0219272)

Fig A

(a)

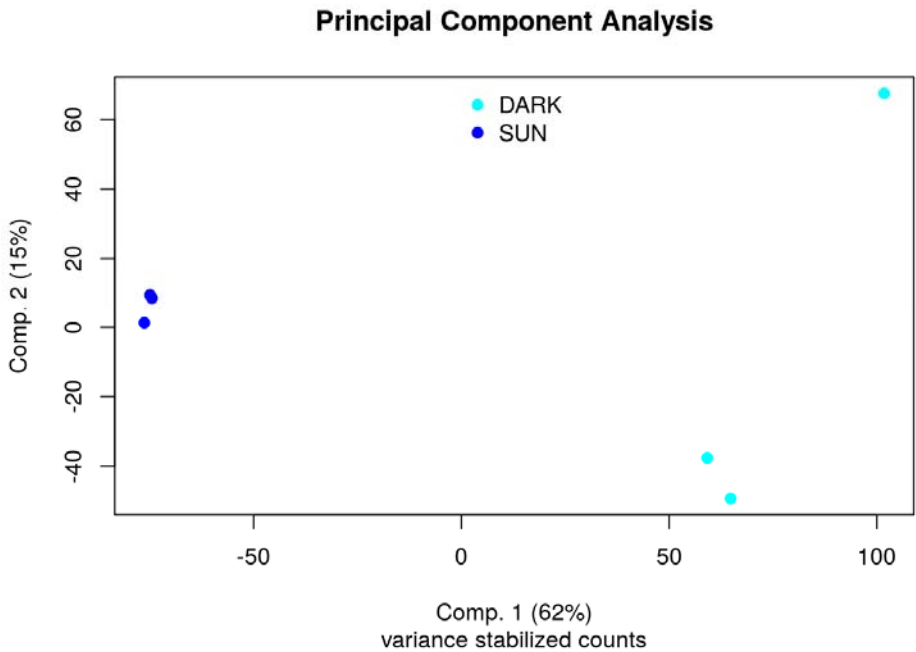

(b)

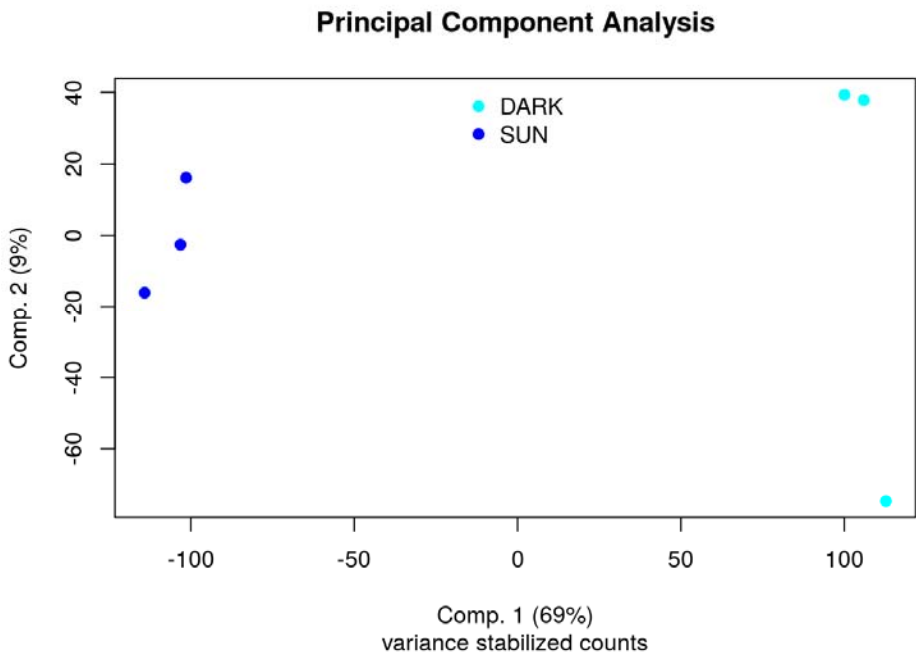

Fig B

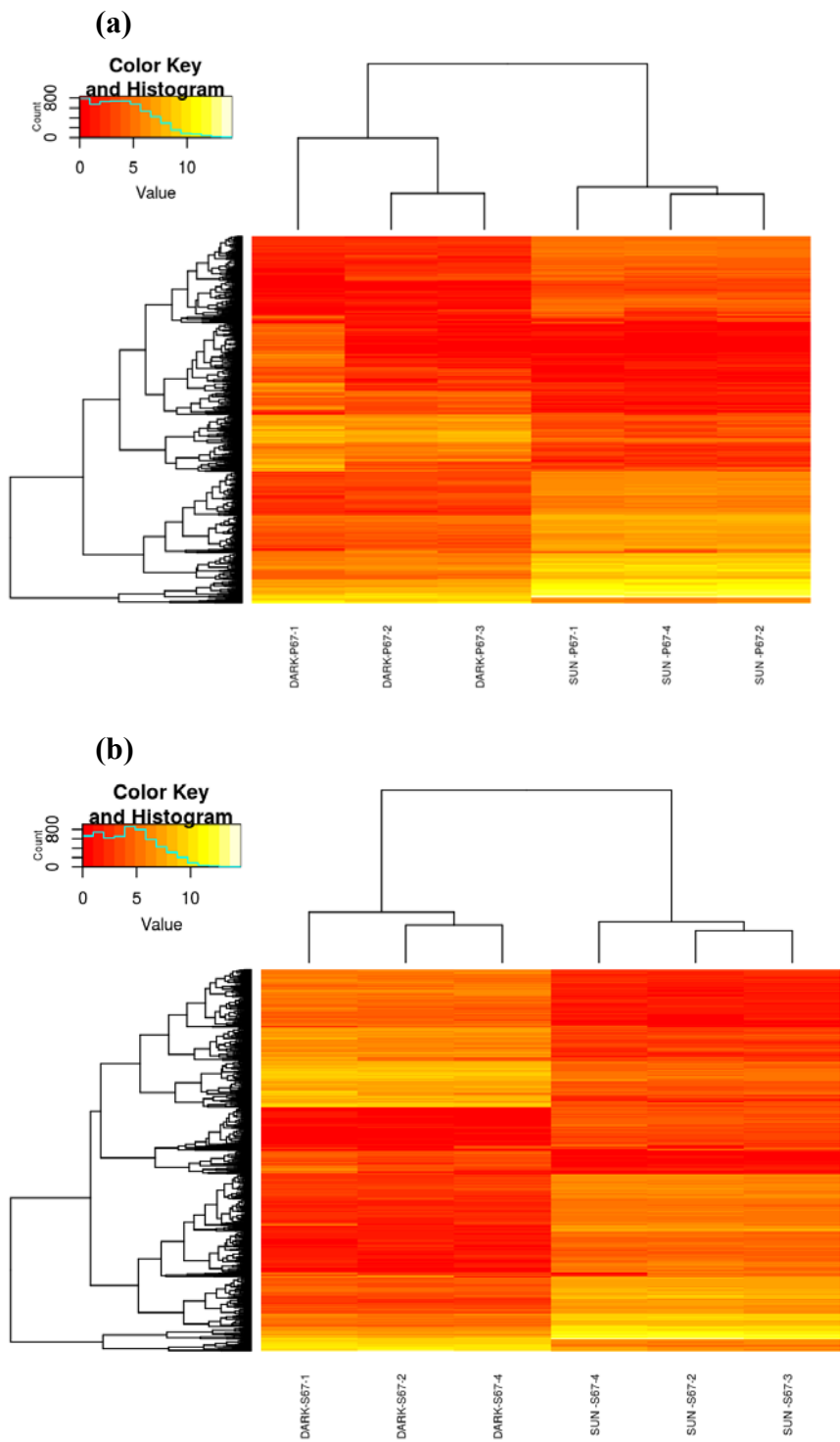

**Fig C**

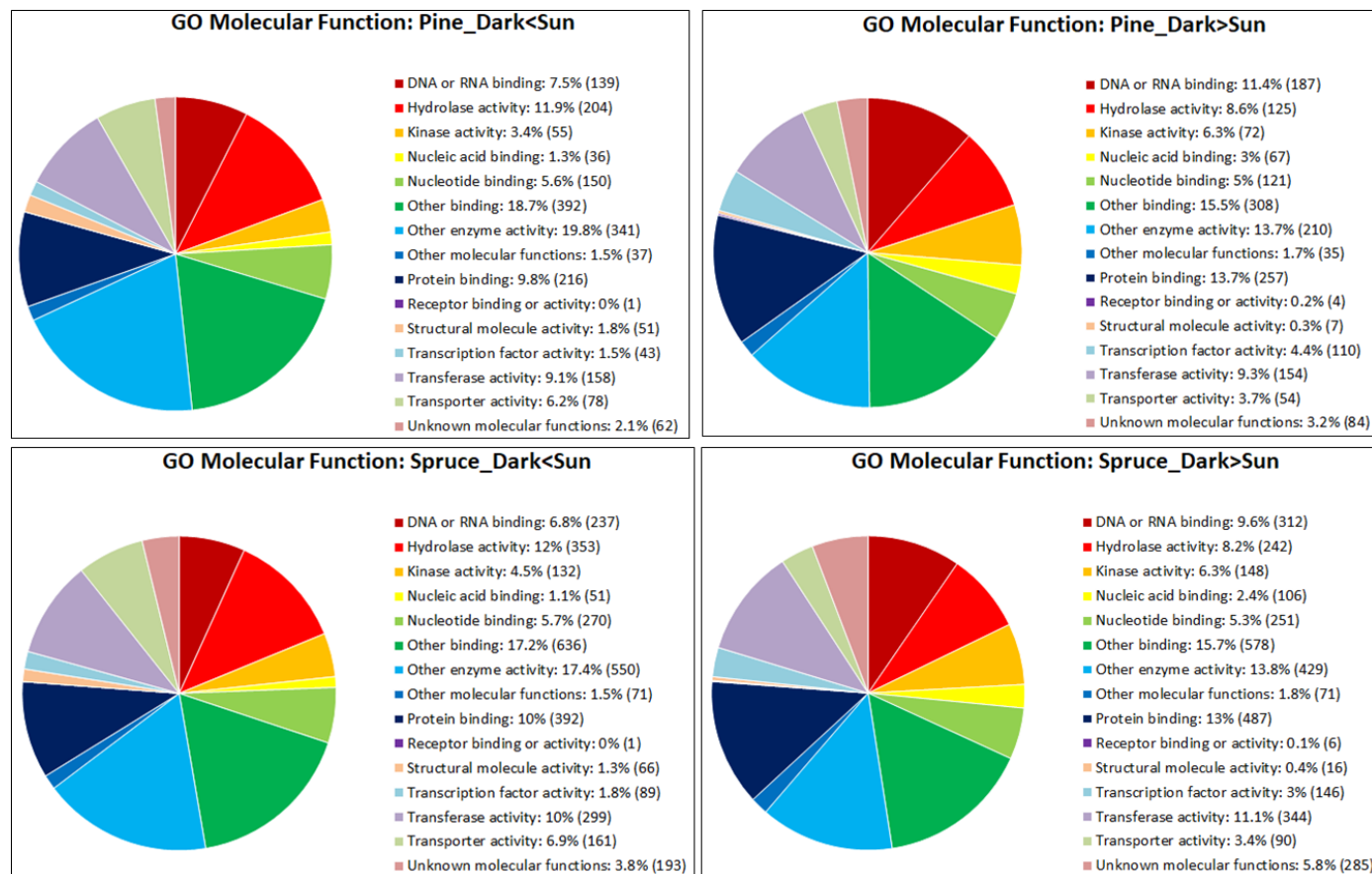

Fig D

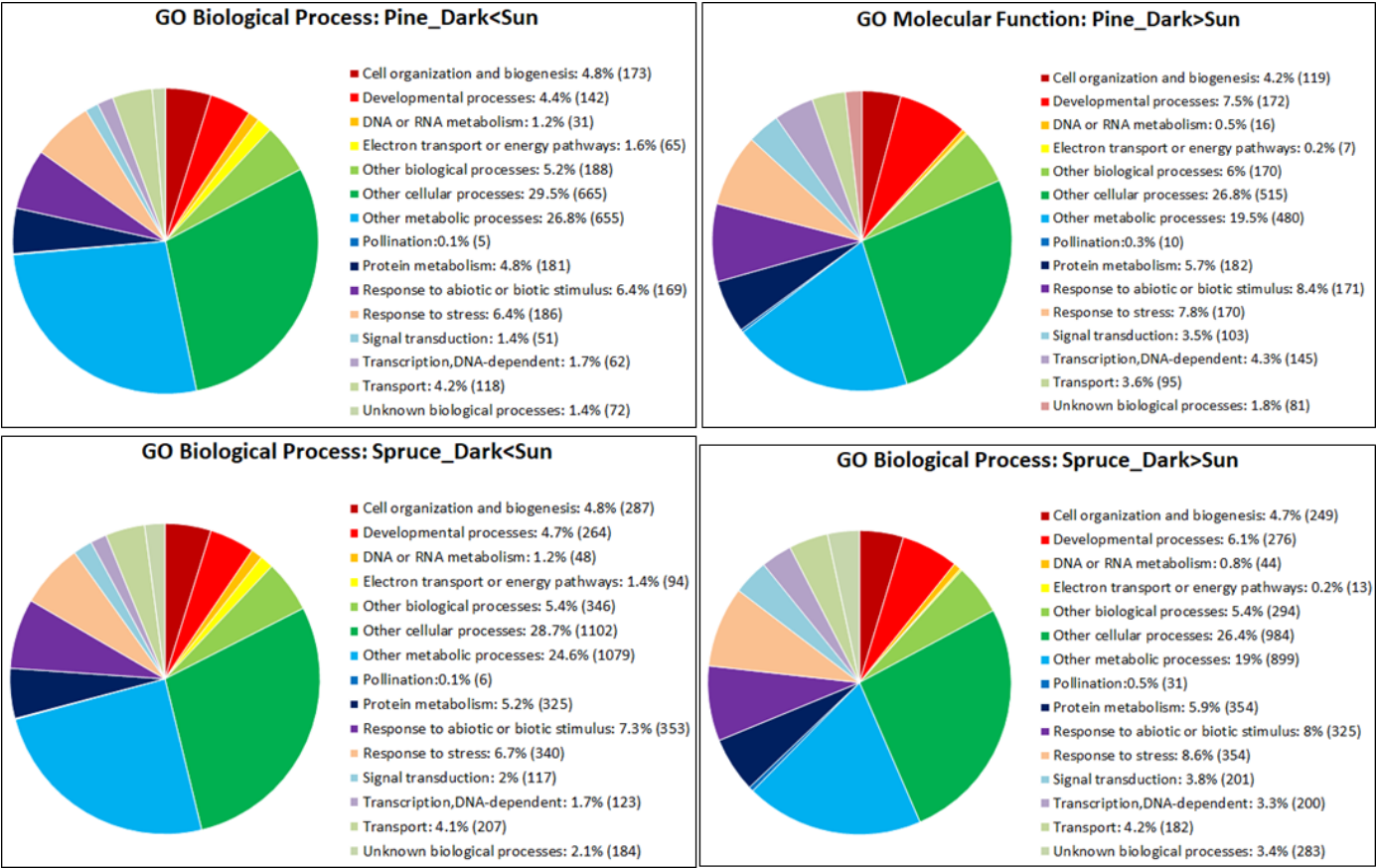

Fig E

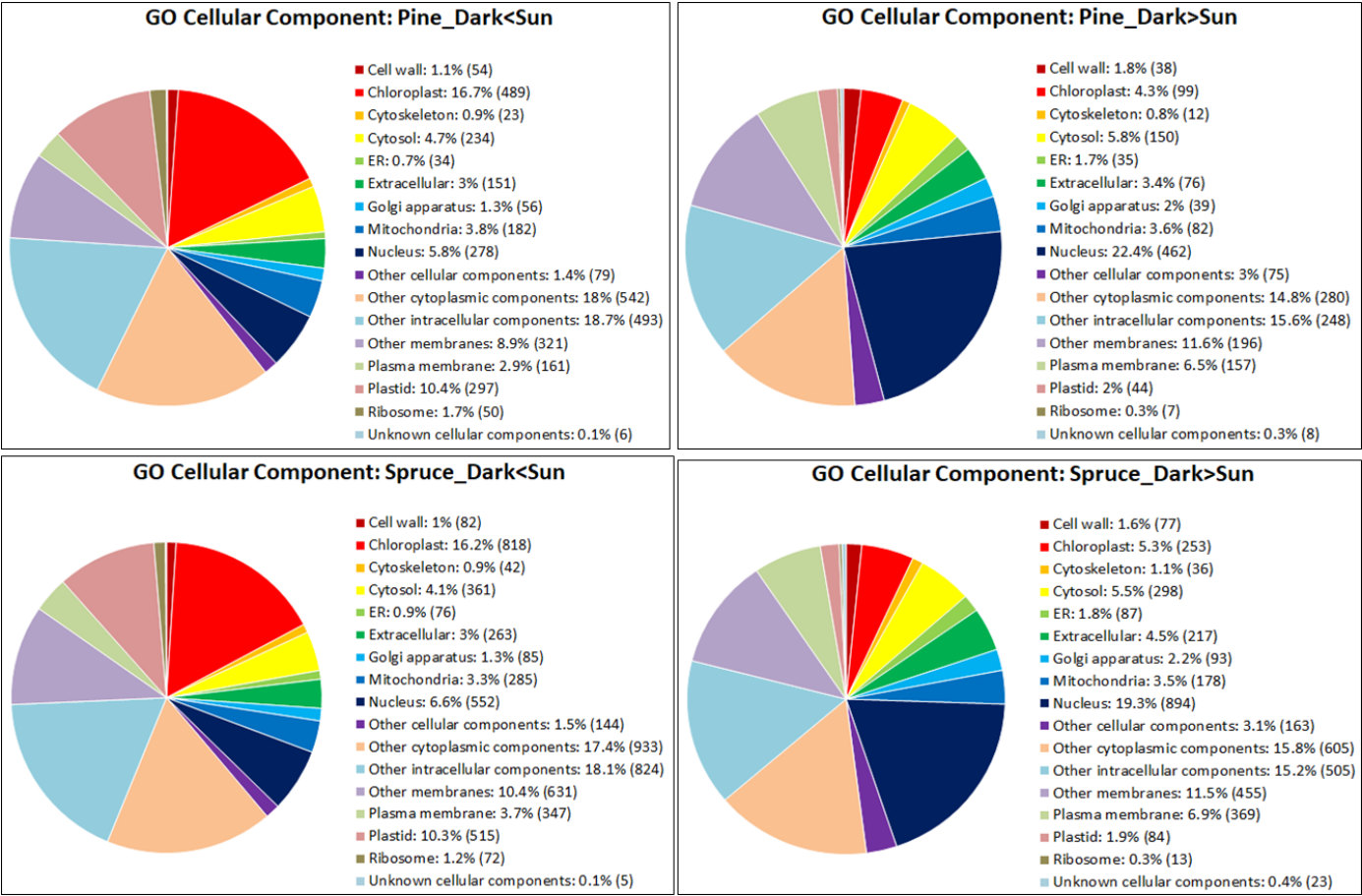

Supplement: S2 File — Figure A PCA based on the entire transcriptome (a) Scots pine (b) Norway spruce Figure B Heatmaps for the entire transcriptome (a) Scots pine (b) Norway spruce Figure C GO Molecular Function: Functional categorization by annotation—pie charts for Scots pine and Norway spruce. Percentage denotes Number of annotations to terms in the GOslim category * 100 / Number of total annotations to terms in the ontology. Number in the parenthesis denotes number of genes in the respective functional category. Figure D GO Biological Process: Functional categorization by annotation—pie charts for Scots pine and Norway spruce. Percentage denotes Number of annotations to terms in the GOslim category * 100 / Number of total annotations to terms in the ontology. Number in the parenthesis denotes number of genes in the respective functional category. Figure E GO Cellular Component: Functional categorization by annotation—pie charts for Scots pine and Norway spruce. Percentage denotes Number of annotations to terms in the GOslim category * 100 / Number of total annotations to terms in the ontology. Number in the parenthesis denotes number of genes in the respective functional category. (PDF) [file pone.0219272.s002.pdf]

Fig A

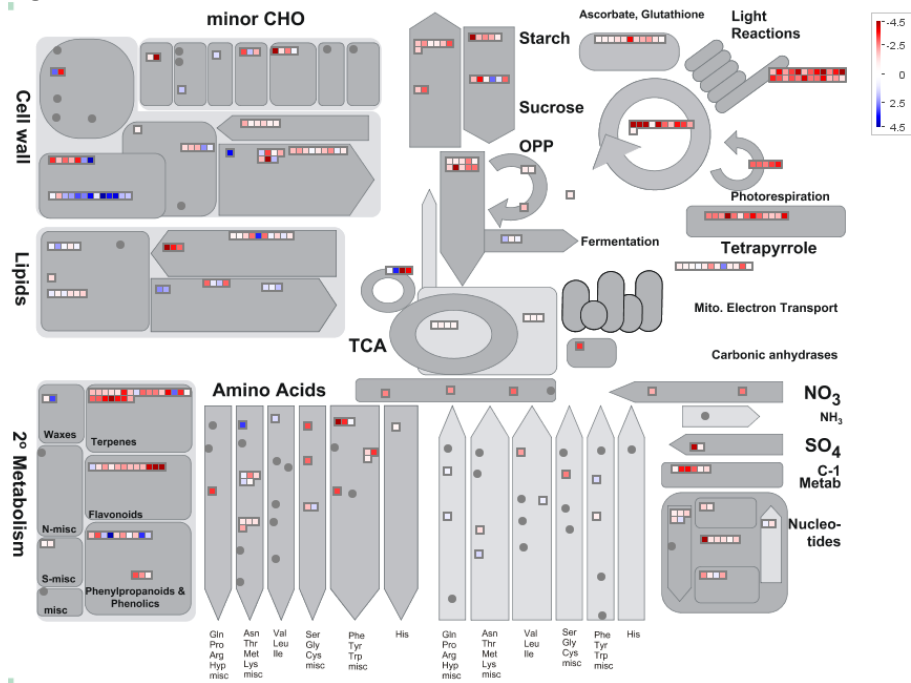

Fig B

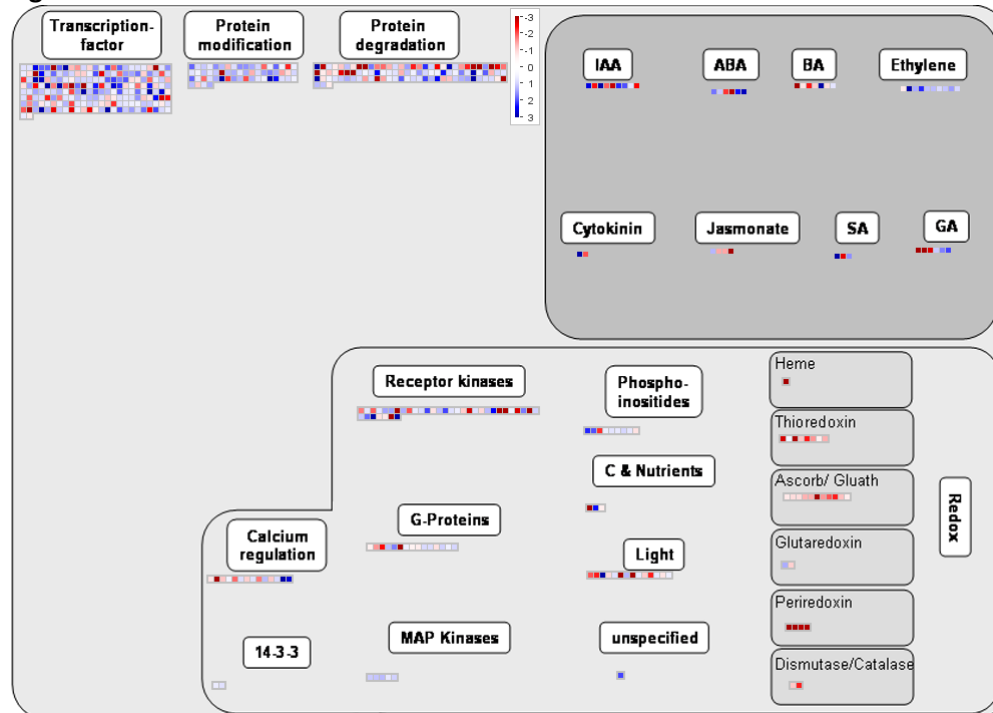

Fig C

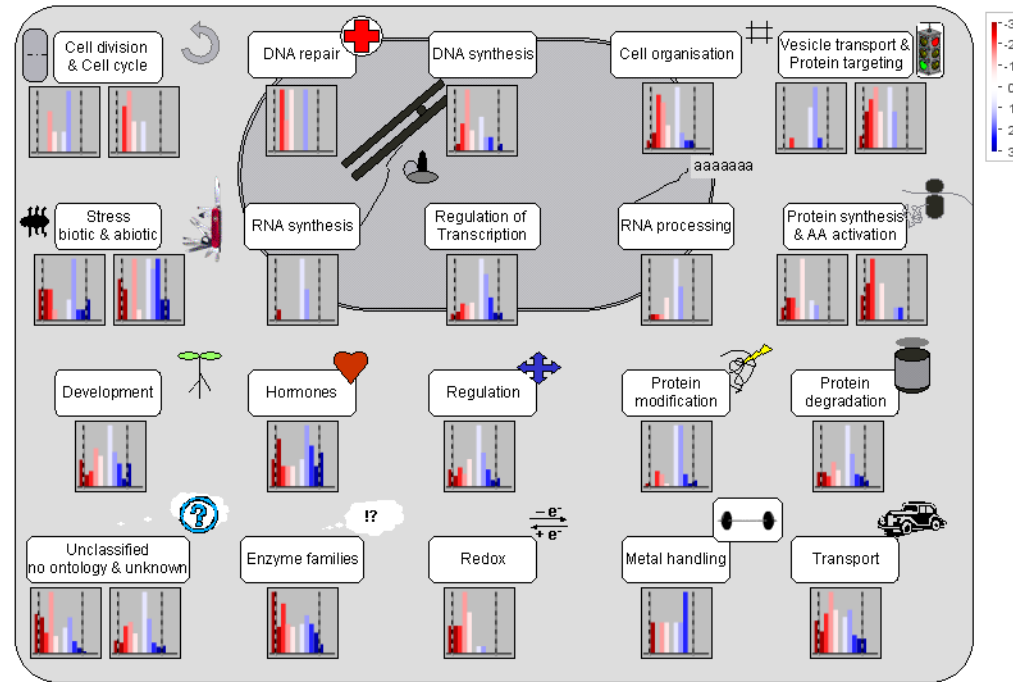

Fig D

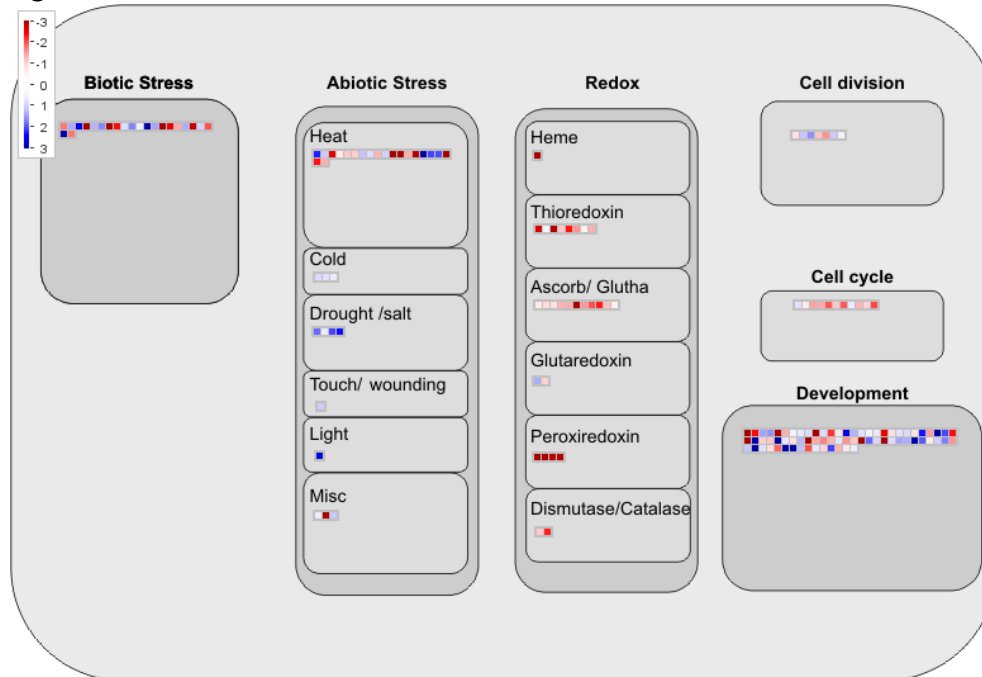

Fig E

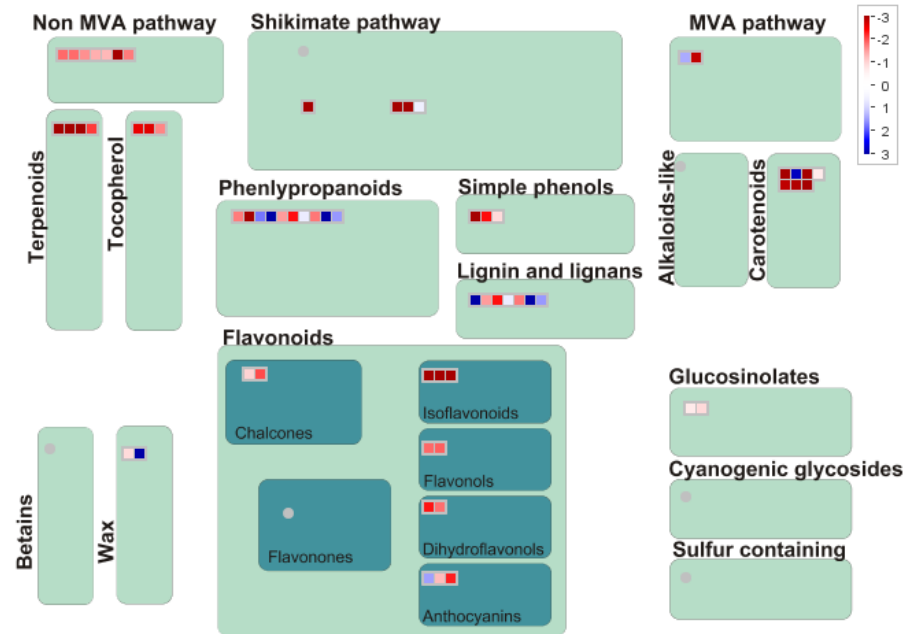

Fig F

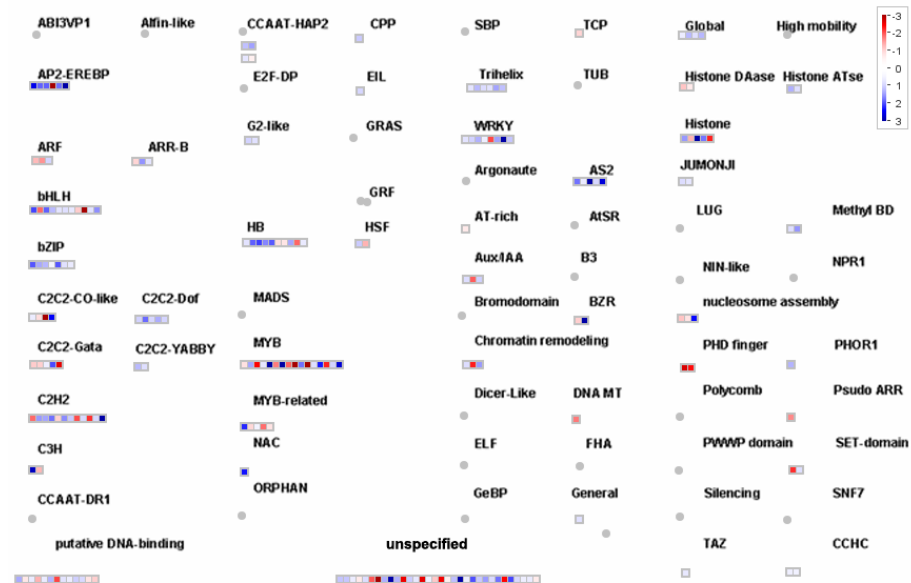

Fig G

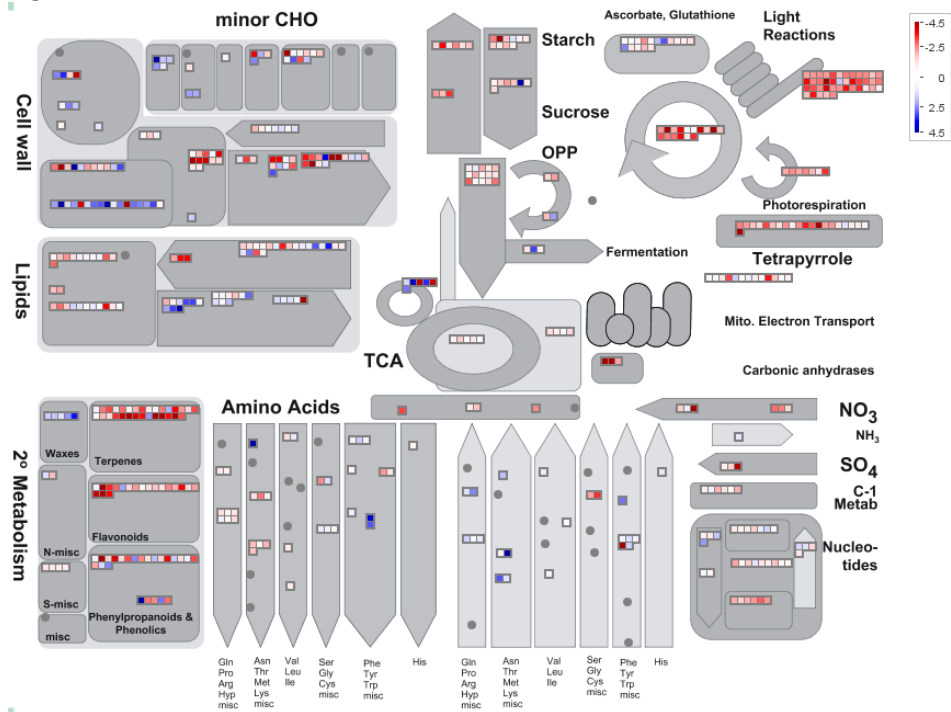

Fig H

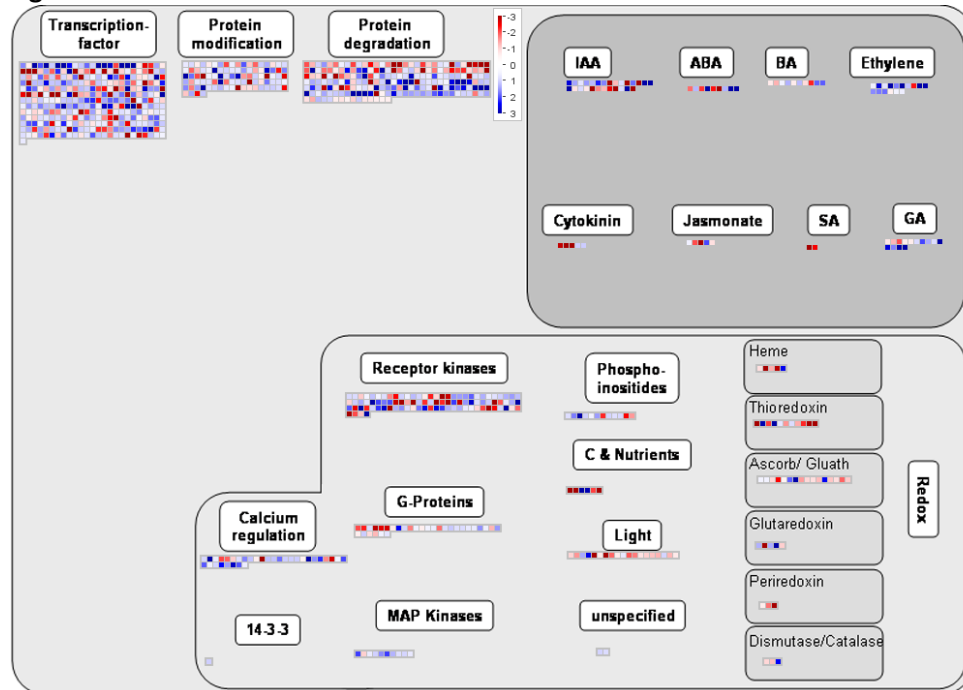

Fig I

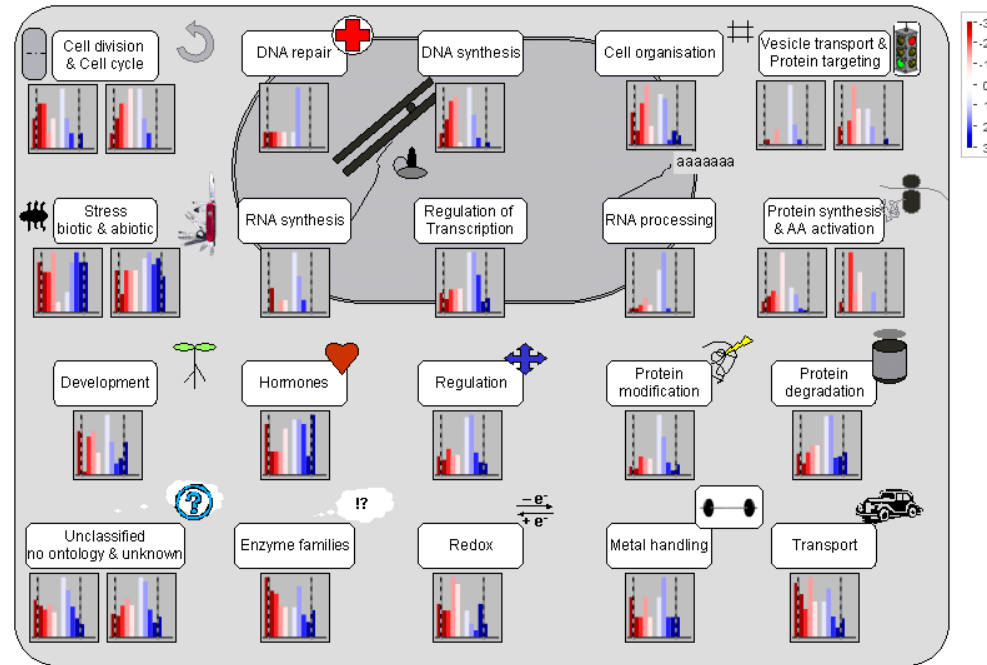

Fig J

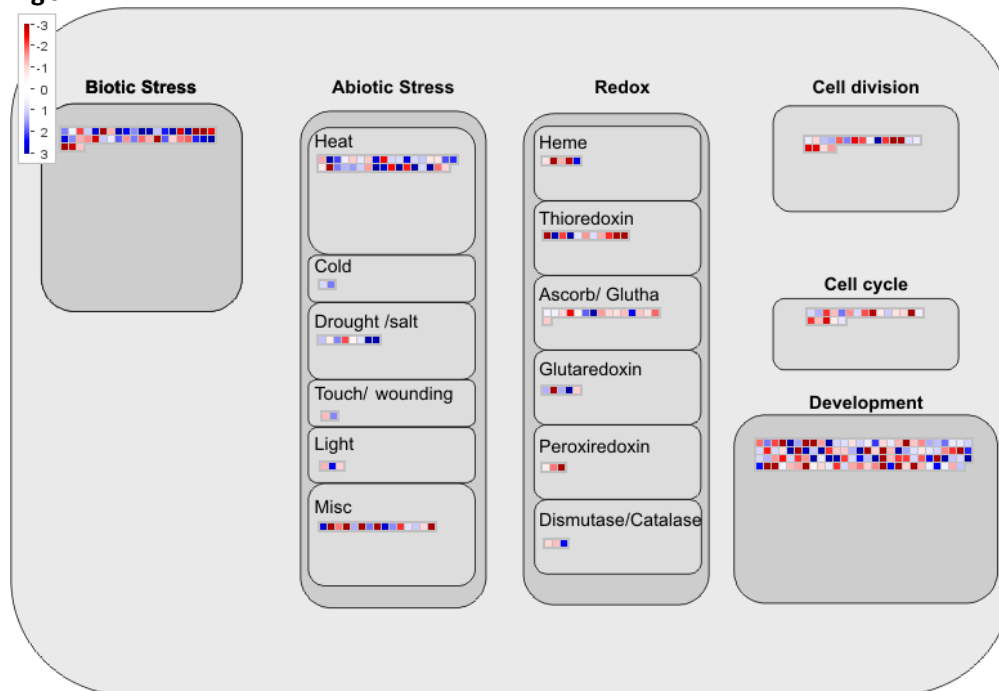

Fig K

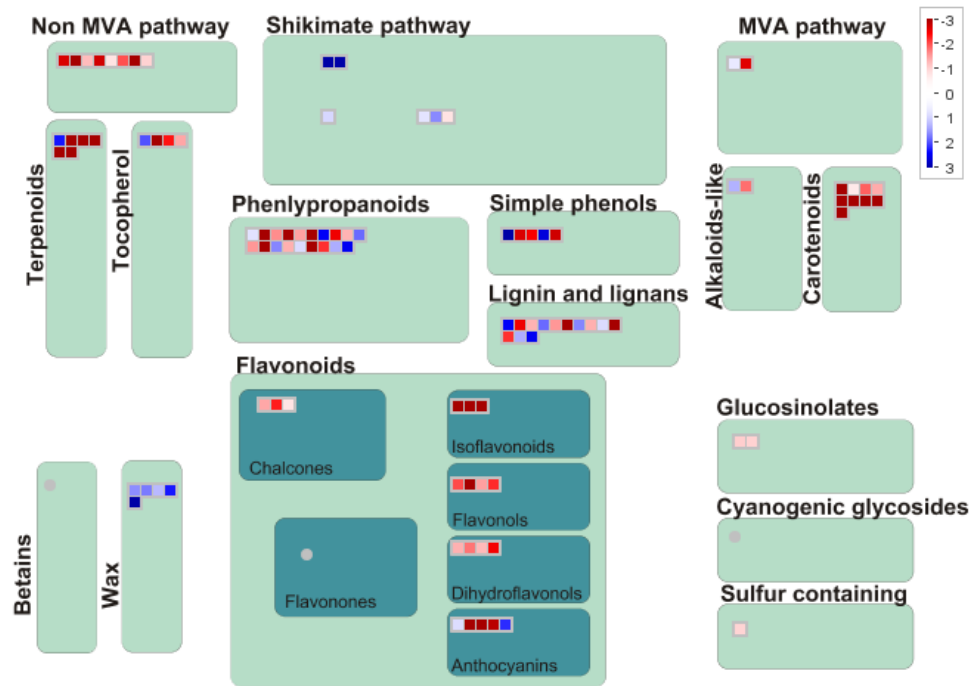

Fig L

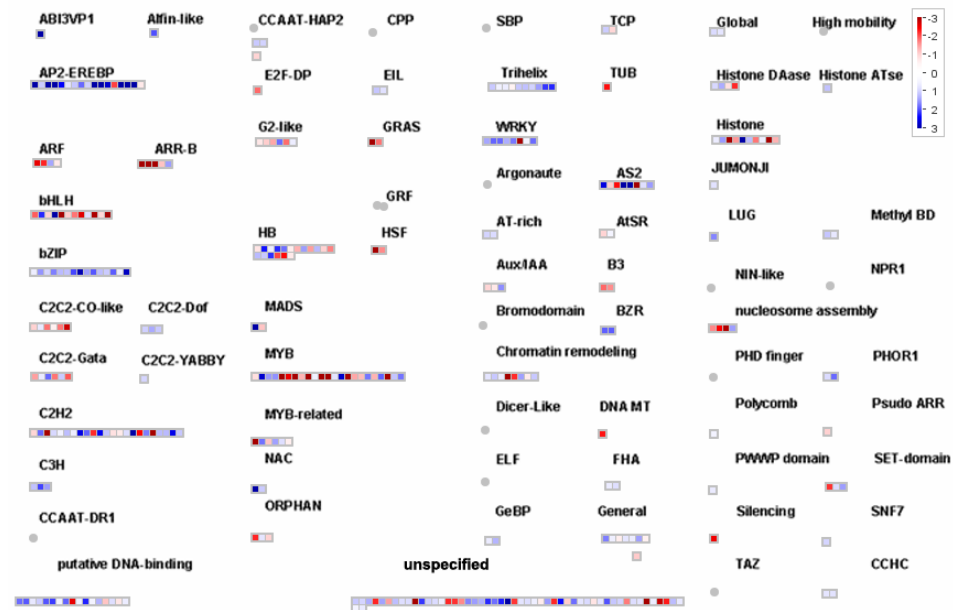

Supplement: S3 File — The metabolic pathway maps constructed using MapMan 3.5.1R2 with the DEGs associated with etiolation/de-etiolation in Scots pine and Norway Spruce. Figure A Metabolism overview in Scots pine Figure B Regulation overview in Scots pine Figure C Cell function overview in Scots pine Figure D Cellular response overview in Scots pine Figure E Secondary metabolism in Scots pine Figure F Transcription in Scots pine Figure G Metabolism overview in Norway Spruce Figure H Regulation overview in Norway Spruce Figure I Cell function overview in Norway Spruce Figure J Cellular response overview in Norway Spruce Figure K Secondary metabolism in Norway Spruce Figure L Transcription in Norway Spruce. (PDF) [file pone.0219272.s003.pdf]
