# Supplementary material for: Global gene expression analysis in etiolated and de-etiolated seedlings in conifers
Source: PLoS One. 2019 Jul 5;14(7):e0219272. doi: 10.1371/journal.pone.0219272 (PMC6611632; doi:10.1371/journal.pone.0219272)
Supplement: S4 File — Protein alignments of CRY1, PIF3 and HY5 from angiosperm and gymnosperm species. (PDF) [file pone.0219272.s004.pdf]

CLUSTAL O(1.2.4) multiple sequence alignment – CRYPTOCHROME 1
Conserved amino acids within conifers and within angiosperms are marked with the box.

```
P.abies             --------MSTNCSIVWFRRDLRVEDNPALAAGVRAGAVIVLYIWSPEEEGQYYPGRVSR      52
P.sylvestris        ------------------------------------------------------------      0
A.thaliana          MSGSVSGCGSGGCSIVWFRRDLRVEDNPALAAAVRAGPVIALFVWAPEEEGHYHPGRVSR      60
G.max               -------MSGGGGSIVWFRRDLRIEDNPALTAGVRAGAVVAVFVWAPEEEGQYYPGRVSR      53
P.trichocarpa       -------MSGGGCSIVWFRRDLRVEDNPALAAGVRAGAVVAVFVWAPEEEGHYYPGRVSR      53
E.grandis           ---------MSGCSIVWFRRDLRVEDNPALAAGVRSGSVLAVFIWAPEEEGAYYPGRVSR      51


P.abies             WWLKQSLAHLDSSLRNLGARLITKRSTNSISAILQVVQATGATQLFYNHLYDPLSLVRDH      112
P.sylvestris        ------------------------------------------------------------      0
A.thaliana          WWLKNSLAQLDSSLRSLGTCLITKRSTDSVASLLDVVKSTGASQIFFNHLYDPLSLVRDH      120
G.max               WWLKNSLAHLHSSLRNLGTPLITKRSTDTLSSLLEVVKSTGATQLFFNHLYDPLSLVRDH      113
P.trichocarpa       WWLKQSLAHLDSSLRSLGTSLVTKRSTDSVSSLLEVVKSTGATQLLFNHLYDPLSLVRDH      113
E.grandis           WWLKHSLAHLDSSLRSLGTCLITRRSTDSVSSLLELVKSTGATQLFFNHLYDPISLVRDH      111


P.abies             KLKQDLSSRGILVRSFNSDLLYEPWEVNDEEGHAFSTFQDYWNKCLNMPYDPLAPLLPPK      172
P.sylvestris        --------------------------INDEEGHAFSTFQAYWNKCLNMPYDPSAPLLPPK      34
A.thaliana          RAKDVLTAQGIAVRSFNADLLYEPWEVTDELGRPFSMFAAFWERCLSMPYDPESPLLPPK      180
G.max               RAKEVLTAQGITVRSFNADLLYEPWEVNDAHGRPFTTFAAFWERCLSMPYDPESPLLPPK      173
P.trichocarpa       RAKEILTAQGITVRSFNADLLYEPWDVNDAQGRPFTTFDTFWERCLSMPFDPEAPLLPPK      173
E.grandis           RAKEVLNAQGINVHSFNADLLYEPWEVNDDQGHPFTTFAGFWEKCLSMPYDPESPLLPPK      171
                                              :.*  *: *: *  :*::**.**:** :******

P.abies             RIVAGEVSNCPSDDINLEDESEKASNALLARAWSPGWSSADKALTAFINGPLLDYAINRQ      232
P.sylvestris        RIVAGEVSNCPSDDLKLEDESEKASNALLARAWSPGWSSADKALTTFINGPLLDYALNRQ      94
A.thaliana          KIISGDVSKCVADPLVFEDDSEKGSNALLARAWSPGWSNGDKALTTFINGPLLEYSKNRR      240
G.max               RIIPGDASRCPSDTLLFEDELEKASNALLARAWSPGWSNANKALTTFINGPLIEYSKNRR      233
P.trichocarpa       RIISGDVSRCPSVTLVFEDESEKGSNALLARAWSPGWSNADRALTTFINGPLIEYSMNRR      233
E.grandis           RIISGDVSRCPSEALVFEDESEKGSNALLARAWSPGWSNADKALTTFVNGPLLEYSKNRR      231
                    :*: *:.*.* :  : :**: **.**************..::***:*:****::*: **:

P.abies             KADSATTSLLSPHLHFGELSVKKVFHLLRIRQVLWANEGNTSGEQSVNLFLKSIGLREYS      292
P.sylvestris        KADSATTSLLSPHLHFGELSVRKVFHLVRIRQVLWANEGNTSGEQSVNLFLKSIGLREYS      154
A.thaliana          KADSATTSFLSPHLHFGEVSVRKVFHLVRIKQVAWANEGNEAGEESVNLFLKSIGLREYS      300
G.max               KADSATTSLLSPHLHFGELSVKKVFHLVRIKQVLWANEGNKAGEESVNLFLKSIGLREYS      293
P.trichocarpa       KADSATTSFLSPHLHFGEVSVRKVFHLVRIKQVLWANEGNRAGEESVNLFIKSIGLREYS      293
E.grandis           KADSATTSFLSPHLHFGEVSVRKVFHLVRIKQVLWANEGNKAGEESVNLFLKSIGLREYS      291
                    ********:*********:**:*****:**:** ****** :**:*****:*********

P.abies             RYLSFNFPSSHEKPLLSHLNYFPWRVDSDYFKSWRQGRTGYPLVDAGMRELWATGWLHDR      352
P.sylvestris        RYLSFNFPSSHERPLLSHLNYFPWRVDRDYFKSWRQGRTGYPLVDAGMRELWATGWLHDR      214
A.thaliana          RYISFNHPYSHERPLLGHLKFFPWAVDENYFKAWRQGRTGYPLVDAGMRELWATGWLHDR      360
G.max               RYISFNHPYSHERPLLGHLKFFPWVVNEGYFKAWRQGRTGYPLVDAGMRELWATGWLHDR      353
P.trichocarpa       RYLSFNHPCTHERPLLGHLKFFPWVVDEGYFKAWRQGRTGYPLVDAGMRELWAAGWLHDR      353
E.grandis           RYMSFNHPYSHERPLLGHLKYFPWVVDEGHFKAWRQGRTGYPLVDAGMRELWATGWLHDR      351
                    **:***.* :**:***.**::*** *: .:**:********************:******

P.abies             IRVVVSSFFVKVLQLPWRWGMKYFWDTLLDADLECDALGWQYISGCLPDGREMDRIDNPQ      412
P.sylvestris        IRVVVSSFFVKVLQLPWRWGMKYFWDTLLDADLECDALGWQYISGCLPDGREMDRIDNPQ      274
A.thaliana          IRVVVSSFFVKVLQLPWRWGMKYFWDTLLDADLESDALGWQYITGTLPDSREFDRIDNPQ      420
G.max               IRVVVSSFFVKVLQLPWRWGMKYFWDTLLDADLESDALGWQYISGSLPDGREIDRIDNPQ      413
P.trichocarpa       IRVVVASFFVKVLQLPWRWGMKYFWDTLLDADLESDALGWQYITGTLPDSREFDRIDNPQ      413
E.grandis           IRVVVSSFFVKVLQLPWRWGMKYFWDTLLDADLESDALGWQYISGTIPDGREFDRIDNPQ      411
                    *****:****************************.********:* :**.**:*******

P.abies             FEGYKFDPAGEYVRRWLPELARLPTEWIHHPWDAPRAVLQAAGVELGSNYPLPIVEISTA      472
P.sylvestris        FEGYKFDPAGEYVRRWLPELARLPTQWIHHPWDAPQAVLQSAGVELGSNYPLPIVEISTA      334
A.thaliana          FEGYKFDPNGEYVRRWLPELSRLPTDWIHHPWNAPESVLQAAGIELGSNYPLPIVGLDEA      480
G.max               FEGYKFDPNGEYVRRWLPELARLPTEWIHHPWNAPESVLQAAGIELGSNYPLPIVGIDAA      473
P.trichocarpa       FEGYKFDPNGEYVRRWLPELARLPTEWIHHPWNAPESVLQAAGIELGSNYPLPIVGIDAA      473
E.grandis           FEGYKFDPNGEYVRRWLPELARLPTEWIHHPWNAPESVLQAAGIELGSNYPLPIVSIDAA      471
                    ******** ***********:****:******:**.:***:**:*********** :. *

P.abies             KERLQEGLSEMWQREADARASLENGTEEGLGESLENANA--SLPD--QMDVDREPVRNNT      528
P.sylvestris        KERLQDALSEMWQREAAARASLENGTEEGLGESLENANA--SLSE--NMDVDREPVRDNT      390
A.thaliana          KARLHEALSQMWQLEAASRAAIENGSEEGLGDSAEVEEAPIEFPRDITMEETEPT----R      536
G.max               KTRLLEALSEMWQQEAASRAAMENGTEEGLGDSSE--SVPAAFPQDMQMEETHEPVRNNP      531
P.trichocarpa       KVRLEEALSEMWQQEAASRAAIENGTEEGLGDSSE--SAPFAFPEDIHMEENHEPVRNNP      531
E.grandis           KVRLQDALSIMWQHEATSRAAIENGTEEGLGDSSE--SAPIAFPQDIQMEENHETMRNNP      529
```

```
                   * ** :.** *** ** :**::***:*****:* *  ..   :     *:  .

P.abies            HLAARSQDDQLVPSISNAVQRAYVGYTLSQAAS-SSPSEQAEVPSNTDSGIASAASSQQF      587
P.sylvestris       GFAARSQDDQLVPSISNMVHRADVTYTLSQAAS-SARSEQAEVPSNRDSGTASAASSQQF      449
A.thaliana         LNPNRRYEDQMVPSITSSLIRPEEDEESSLNLRNSVGDSRAEVPRNMVNTNQAQQ---R-      592
G.max              LPVARRYQDQMVPSITSSLLRVEEEETSSDLRHSAEESSRAEVPVTANAQQNV-G---V-      586
P.trichocarpa      PATNRRYEDQMVPSMTSSFLRIEDEETS-DVRNST-GDGRAEVPRDVNVNQEPRR---D-      585
E.grandis          PGPVHRYEDQMVPSITSLVRADEEETSS-GIQNSI-GDTRAEVPANVNLNEEPRR---D-      583
                       :  :**:***::  .                  . :****

P.abies            LGQSTWSMTRGLIEDL--PNVTNDYPQMQVLDEQNESHSTADSSSILRGRDDMSSGGIVP      645
P.sylvestris       LGQSTWSMTRGLIEDL--PNVISHYPQMQALDEQSESHSTADSSSILRVRDDTSSGGIVP      507
A.thaliana         ------RAEPAS---NQVTAMIPEFNIR-IVAESTE-DSTAESSSS---GRRERSGGIVP      638
G.max              ------TLNERMLQ-TTNRNAQTQYNTTMELRNVAE-DSAVESSSG---TRRERDGGVVP      635
P.trichocarpa      ------ALNQGFVQTVRNNTALSPFNISRGLTN-VE-DSTAESSSS---GRRERDGGIVP      634
E.grandis          ------TLNEGVFQQLRPNNTQPMYNFSPGLHNDAE-DSTAESSST---GRRERDGGVVP      633
                                           :     : :  *  .*:.:***         .**:**

P.abies            VWSQSLSQSC--------------------------------------------------      655
P.sylvestris       VWSQSLSGHSQQFPEEGTVPGTAESSLQQQLHNHSMGTWRHGHNQGAVQMSKPVCSEFNF      567
A.thaliana         EWSP---GYSEQFPSEENGIGGGSTTSSYLQNHHEILNWRRLSQTG--------------      681
G.max              VWSPPASSYSEQFVGEENGITNSSSFLQRHPQSHQMLNWRQLPQTG--------------      681
P.trichocarpa      VWSPPTSSYSEQFVGDDNGIGATSSYLQRHPQSHQIINWRRLSQTG--------------      680
E.grandis          VWSPSTSSYSDQIAGDENGIGNGSAFLQRHPQSHQIINWRRLSQTG--------------      679
                    **      .

P.abies            -----  655
P.sylvestris       KYAVL  572
A.thaliana         -----  681
G.max              -----  681
P.trichocarpa      -----  680
E.grandis          -----  679
```

Sequence details

P.abies	MA_10428291p0010 (http://congenie.org/)
P.sylvestris	AFV79203.1 (https://www.ncbi.nlm.nih.gov/)
A.thaliana	AT4G08920.1 (https://www.arabidopsis.org)
G.max	Glyma.04G101500.1 (https://phytozome.jgi.doe.gov/)
P.trichocarpa	Potri.005G164700.2 (https://phytozome.jgi.doe.gov/)
E.grandis	Eucgr.F00326.1 (https://phytozome.jgi.doe.gov/)

CLUSTAL O(1.2.4) multiple sequence alignment – PIF3

```
A.thaliana      MPLFELFRLTKAKLESAQDRNPSPPVDEVVELVWENGQISTQSQSSRSRNIPPPQANSSR    60
P.taeda         ------------------------------------------------------------    0
P.abies         ------------------------------------------------------------    0
P.sylvestris    ------------------------------------------------------------    0


A.thaliana      AREIGNGSKTTMVDEIPMSVPSLMTGLSQDDDFVPWLNHHPSLDGYCSDFLRDVSSPVTV    120
P.taeda         -----------------------------------MSHYRPSLTSFIPK----AS-----    16
P.abies         ------------------------------------------------------------    0
P.sylvestris    ------------------------------------------------------------    0


A.thaliana      NEQESDMAVNQ--TAFPLFQRRK----DGNESAPAASSSQYNGFQSHSLYGSDRARDLPS    174
P.taeda         ------LQVKTYIFSFSFYTLIHCYGFAGPRRGASTSRLIQTGFPPSR--------QKHK    62
P.abies         ------------------------------------------------------------    0
P.sylvestris    ------------------------------------------------------------    0


A.thaliana      QQTNPDRFT------QTQEPLITSNKPSLVNFSHFLRPATFAKTTNNN-LHDTKEKSPQS    227
P.taeda         DQKTP-HFALRIMAQKCSEPGFLVNNSALWDDED-----ALNRNYNYNYYTDLWGGLPLS    116
P.abies         ------------MAHKCLEPSFLVHNPVLWDDED-----ALNRNYNYNYYTDLWGGLPLS    43
P.sylvestris    ------------MAQKCSEPGFLVNNSALWDDED-----ALNRNYNYNYYTDLWGGLPFS    43
                               :  ** :  ::  * : ..     :: :. * *   *     * *

A.thaliana      PPNVFQTRVLGAKDSEDKVLNESVASATPKDNQKACLISEDSCRKDQESEKAVVCS---S    284
P.taeda         SS-AP--ARTSAEDSRPKVVAASEAVE--SDYG--RVNK--N-NNTVSADTQLSCETAAT    166
P.abies         SS-VATTARTSAEDSRPEVVAVSEALE--SDYG--RLNN--NNNNNVSADTQLSCETAAT    96
P.sylvestris    SS-APATARTSAENSRPKVVAASEAVE--SDYG--RVNN--K-NNNVSADTQLSCETAAT    95
                   .      .*::*. :*:  * *    .*     : .  . .:  .::. : *.   :

A.thaliana      VGSGNSLDGPSESPSL---SLKRKHSNIQDIDCHSEDVEEESGDGRKEAGPSRTGLGSKR    341
P.taeda         SSSGGSMSSPSKSNEVIKSSKNKVLMGTKDIECQSQ------------------------    202
P.abies         SSSGGSMSSPSNGNKVIKSSKKKVLMGTKDIECQSQKAQEDSGENFKQCST--GTSSSKR    154
P.sylvestris    SSSGGSMSSPSKSNEVIKSSKNKVLMGTKDIECQSQKAREDSGENFKQCST--GTS-SKR    152
                 .**.*:..**:. .:   * ::   . :**:*:*:

A.thaliana      SRSAEVHNLSERRRRDRINEKMRALQELIPNCNKVDKASMLDEAIEYLKSLQLQVQIMSM    401
P.taeda         ------------RRRNRINEKMKALQNLIPNSNKTDKASMLDEAIEYLKKLQLQVQMLSA    250
P.abies         SRAAEVHNLSEKRRRNRINEKMKALQNLIPNSNKTDKASMLDEAIEYLKKLQLQVQMLSA    214
P.sylvestris    SRAAEVHNLSEKRRRNRINEKMKALQNLIPNSNKTDKASMLDEAIEYLKKLQLQVQMLSA    212
                            ***:******:***:****.**.**************.******::*

A.thaliana      ASGYYLPPAVMFPP----GMGHYPAAAAAMA----MGMGMPYAMGLPDLSRGGSSVNH--    451
P.taeda         RSGIDLSSMRWLAQMPHLQIQQMPKACMAIDQHAGVPISMPVGSGLINTNQGSEKRPLPL    310
P.abies         RSGIDISSMRWLAQMPHLQIQQMPKACMTTDQHAGVSISMPVGSGLMNTNQGSEKRPLPL    274
P.sylvestris    RSGIDLSSMRWLAQMPHLQIQQMPKARMAIDQHAGVPISMPAGSELINTNQGSEKRPLPL    272
                 **  :     :       : : * *  :      : :.** .  * : .:*...

A.thaliana      GPQFQVSGMQQQPVAMGIPR--VSGGGIFAGSSTIGNGSTRDLSGSKDQTTTNNNSNLKP    509
P.taeda         HDLYKSGALCNTAIPINLSSVRIDDHHSCKMDHPQGHFTLPQLPT----TTQEVHSAL-S    365
P.abies         HDLYISGALGNTAIPINLPSARIDDHQSCKMDRPQGHFTLPQLPT----TTQEVHSAL-S    329
P.sylvestris    HDLYNSGALGNTAIPINLSSVRIDDHHSCKMDHPQGHFTLPQLPT----TTQEVHSAL-S    327
                   :  ..: :  : :.:    :..      .   *: :  :*      ** : :* *

A.thaliana      IKRKQGSSDQFCGSS 524
P.taeda         LQEK----------- 369
P.abies         LQEK----------- 333
P.sylvestris    LQEK----------- 331
                ::.*
```

Sequence details

P.abies MA_26114p0010 (http://congenie.org/)
P.sylvestris PSY00020194 (https://bioinformatics.psb.ugent.be/plaza/)
A.thaliana AT1G09530.2 (https://www.arabidopsis.org)
P.taeda PITA_000009665 (http://congenie.org/)

CLUSTAL O(1.2.4) multiple sequence alignment – HY5

```
P.abies            MQAIAPDFSG----SATGDKLITSSSGDPHEEAMNECVNESDDDIRRVPEMGVQTGFVM-       55
P.taeda            MQGTAPDLSG----SATGDKFFTSSSGDLHEEARNECVNESDDDIRRVPEMGVQTGVVV-       55
A.thaliana         MQEQATSSLAASSLPSSSERS---SSSAPHLE--IKEGIESDEEIRRVPEFGGEAVGKET       55
P.sylvestris       MQDTAASTST--QHQSTSEKSSSSAAPAQFRQ--AKDAIESDDDIRRVPEMGGMQAGPST       56
                   **  * .        ::.::    ::   . :   :   ***::******:*

P.abies            --HSDLAVKTESERASASGGSQRRRGRTPADKEHKRLKRLLRNRVSAQQARERKKAYLNE       113
P.taeda            --NSDLVVKTESERGLSGSGSQRRRGRTPADKEHKRLKRLLRNRVSAQQARERKKAYLNE       113
A.thaliana         SGRESGSATGQERTQATVGESQRKRGRTPAEKENKRLKRLLRNRVSAQQARERKKAYLSE       115
P.sylvestris       CVPMRLD----NPQPSTGVVAHRKRGRAPADKEHKRLKRLLRNRVSAQQARERKKAYLND       112
                              .    :   ::*:***:**:**:************************.:

P.abies            LETKANELQQKNSELEERVSTLQNENFMLRQVLKNMTAKNKGGGFVEAEG---      163
P.taeda            LETKAKELQQRNSELEERVSTLQNENYMLRQVLKNMTAKNKGGGVGEVEG---      163
A.thaliana         LENRVKDLENKNSELEERLSTLQNENQMLRHILKNTTGNKRGGGGGSNADASL      168
P.sylvestris       LETRVKEIEHKNSELEERLSTLQNENHMLRQILKNTTMKKKGSGNSGAET---      162
                   **.:.::::::*******:******* ***::*** * :::*.*
```

Sequence details

```
P.abies            MA_41006p0010 (http://congenie.org/)
P.sylvestris       PSY00021926 (https://bioinformatics.psb.ugent.be/plaza/)
A.thaliana         AT5G11260.1 (https://www.arabidopsis.org)
P.taeda            PITA_000075685 (http://congenie.org/)
```
